# Migration of styrene oligomers from food contact materials: in silico prediction of possible genotoxicity

**DOI:** 10.1007/s00204-022-03350-x

**Published:** 2022-08-13

**Authors:** Elisa Beneventi, Christophe Goldbeck, Sebastian Zellmer, Stefan Merkel, Andreas Luch, Thomas Tietz

**Affiliations:** 1 Department of Chemical and Product Safety, German Federal Institute for Risk Assessment (BfR), Max-Dohrn-Strasse 8-10, 10589 Berlin, Germany; 2 Chemical and Veterinary, Analytical Institute Muensterland-Emscher-Lippe (CVUA-MEL), 48147 Münster, Germany

**Keywords:** Styrene oligomers, NIAS, Migration, Genotoxicity prediction, In silico tools, (Q)SAR, Food contact materials

## Abstract

Styrene oligomers (SO) are well-known side products formed during styrene polymerization. They consist mainly of dimers (SD) and trimers (ST) that have been shown to be still residual in polystyrene (PS) materials. In this study migration of SO from PS into sunflower oil at temperatures between 5 and 70 °C and contact times between 0.5 h and 10 days was investigated. In addition, the contents of SD and ST in the fatty foodstuffs créme fraiche and coffee cream, which are typically enwrapped in PS, were measured and the amounts detected (of up to 0.123 mg/kg food) were compared to literature data. From this comparison, it became evident, that the levels of SO migrating from PS packaging into real food call for a comprehensive risk assessment. As a first step towards this direction, possible genotoxicity has to be addressed. Due to technical and experimental limitations, however, the few existing in vitro tests available are unsuited to provide a clear picture. In order to reduce uncertainty of these in vitro tests, four different knowledge and statistics-based in silico tools were applied to such SO that are known to migrate into food. Except for SD4 all evaluated SD and ST showed no alert for genotoxicity. For SD4, either the predictions were inconclusive or the substance was assigned as being out of the chemical space (out of domain) of the respective in silico tool. Therefore, the absence of genotoxicity of SD4 requires additional experimental proof. Apart from SD4, in silico studies supported the limited in vitro data that indicated the absence of genotoxicity of SO. In conclusion, the overall migration of all SO together into food of up to 50 µg/kg does not raise any health concerns, given the currently available in silico and in vitro data.

**Supplementary Information:**

The online version contains supplementary material available at 10.1007/s00204-022-03350-x.

## Introduction

Styrene oligomers (SO) are chemicals formed during PS manufacturing. PS is a versatile thermoplastic polymer employed in many materials and used in various consumer products. One of its main applications is food packaging, where 50–60% of the PS production volume are used (Nakai et al. 2014). In general, residual substances can be present in the PS materials as Intentionally Added Substances (IAS), including starting monomers or additives that, being well characterized and regulated by the regulation (EU) No 10/2011 (EC 2011) on plastic materials and articles intended to come into contact with food, do not pose any health risk. Non-Intentionally Added Substances (NIAS) could also be present in the material and, since NIAS are not (specifically) regulated by the cited plastics regulation, additional risk assessment is needed. NIAS are defined in Article 3(9) of the regulation (EU) No 10/2011 as “an impurity in the substances used or a reaction intermediate formed during the production process or a decomposition or reaction product”. In Article 6(4) and Article 19 of this regulation it is outlined that NIAS—despite not being picked up in the positive list of the regulation—may be present in food contact materials (FCM) made of plastics and that safety with respect to human health [compliance with Article 3 of the framework regulation (EC) No 1935/2004] “shall be assessed in accordance with internationally recognized scientific principles on risk assessment” by the business operator. These principles are being discussed, e.g., in EFSA (2016).

SO (c.f. Table 1) are typical NIAS. The main groups are styrene dimers (SD) and styrene trimers (ST). The first identification and quantification was performed in food packaging and later the migration into food simulant (Kawamura et al. 1998a) and instant food (Kawamura et al. 1998c) was measured.

**Table 1:**
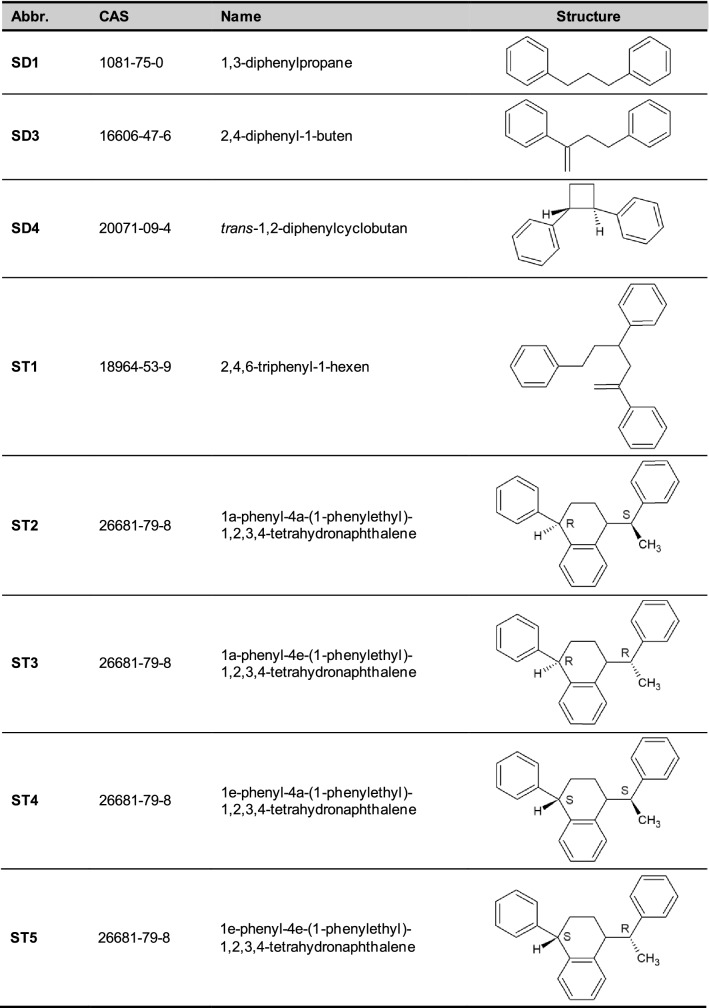
Abbreviations, identifier and chemical structures of styrene dimers (SD) and trimers (ST). Axial and equatorial symmetry is indicated by letters “a” or "e”

SD and ST are the most relevant SO that need to be assessed. Due to their molecular weight below 1000 Da the gastrointestinal uptake of these compounds seems likely (EFSA 2008). In 2016, the official German control laboratory CVUA-MEL has performed migration tests on different SO (Table 1) from twelve commercially available PS FCM. A summed up migration level of up to 51 µg/kg food simulant was found at migration conditions of 50% ethanol for 2 h at 70 °C (Funke et al. 2018). In 2016, BfR has did not find any health risks associated to SO after evaluating the scientific literature on genotoxicity, endocrine activity and developmental toxicity available at this time (BfR 2016). Another risk assessment of SO from non-expanded PS has been published recently (Gelbke et al. 2019). Different approaches were used in this evaluation, including the TTC concept and the FACET methodology (Oldring et al. 2009) for exposure estimation. Gelbke et al. (2019) concluded that SO pose a low risk for consumers.

Both risk assessments took into consideration the same few available genotoxicity studies pointing to the absence of genotoxicity. In fact, to date there are only two studies concerning in vitro genotoxicity of SO. In 1990, Griffol et al. (1990) performed an Ames test using the *Salmonella typhimurium* strain TA98 and a metabolic activation system. Unfortunately, the authors did not publish the complete set of data needed to be in line with the OECD Test Guideline (TG) 471. According to OECD TG 471, the substance should be tested in a specific set of several bacterial strains as well as in the presence and absence of metabolic activation. Later, Nakai et al. (2014) performed an Ames (OECD TG 471) and a chromosomal aberration test (OECD TG 473) with SO extracted from PS FCM. Although the assays were performed according to the corresponding OECD TG, many limitations apply. Most important, the concentrations of some of the SO, especially the SD, in the testing solution were very low. Hence, the negative result found is not considered sufficient to firmly rule out a genotoxic concern for all individual SO investigated.

In order to increase confidence in the conclusion that SO are non-genotoxic, alternative approaches, like in silico methodologies, can be used. REACH, the European regulation on chemicals (Regulation (EC) No 1907/2006), has encouraged their use to generate data since 2006 and they have been increasingly applied in many sectors (EMA 2018). Also EFSA has promoted the use of non-testing methods in a guidance document on the use of the WoE approach (EFSA 2017). According to this, all data that allow for the reduction of uncertainties should be taken into consideration and specifically, the combination of evidence from testing and non-testing methods, properly weighted and integrated, is suitable to achieve reliable conclusions.

Prediction of genetic toxicity through non-testing methods is based on structural alerts that can be directly correlated to the biological activity of the target chemical. Main methodologies include (quantitative) structure activity relationships (QSAR), grouping and read-across methods.

The present work aims to reduce the uncertainties derived from the limited in vitro genotoxicity data on SO using in silico approaches. In addition, data from the literature and from our own work on the contents in and the levels of SO migrating from PS FCM are summarized.

## Materials and methods

### Oligomer analysis

Funke et al. (2018) developed an online LC–GC method coupled to triple quadrupole mass spectrometry (QqQ) with which styrene oligomers (dimers and trimers) as well as styrene and methyl styrene in foods and food simulants can be identified and quantified. With this method, very low detection limits between 0.000 001 mg/kg and 0.000 060 mg/kg (1–60 ng/kg) can be achieved in fatty foods and edible oils. Exactly this examination method is also used by the CVUA-MEL.

With the LC pre-separation, interfering matrix components, such as triglycerides, can be separated from the SO. Compared to conventional GC–MS/MS analytics, online LC-GC-QqQ has the advantage that the food simulant edible oil can be examined in the routine. Migration studies by other working groups were mostly carried out with ethanol. However, in particular 95% ethanol leads to a significant overestimation of migration, because this simulant migrates into the plastic material thereby causing its swelling. In contrast to 95% ethanol, edible oil represents real food. The migration results obtained with edible oil are therefore advantageous for the respective exposure assessment.

After the styrene dimers and styrene trimers have been fractionated by normal phase liquid chromatography (NPLC), the SO are transferred online to gas chromatography (GC) directly in the LC mobile phase. In a first step on an unpacked GC column, the LC solvent and interfering substances are removed from the GC via an open valve at the end of the unpacked GC column. In course of this, concentrations of analytes are further increased, resulting in higher sensitivity of the method. After closing the valve, the purified and concentrated fractions are transferred to the packed GC capillary for chromatographic separation and subsequent quantification of the SO via triple quadrupole mass spectrometry (QqQ).

### Data compilation

Not all cited work published the sum of the extracted or migrated SO. In these cases, the sum in Tables 2, 3 and 4 was calculated based on the data given for the individual SO. In some studies, additional SO in comparison to those regarded in this work were measured. Therefore, the sum values reported in this work were adjusted accordingly. In some cases, studies were only available in Japanese. In these cases, information was taken from the English abstract and tables.

**Table 2:** Migration (in mg/kg) of styrene dimers (SD) and styrene trimers (ST) from PS materials into food simulants

Material	Food simulism (EtOH if not specified)	Dimers			Trimers					Sum		References
		SD1	SD3	SD4	ST1	ST2	ST3	ST4	ST5	ST2, ST3, ST4, ST5	All oligomers	
EPS	Sunflower oil, 5 °C, 3 d	ND (< 0.000001)	ND (< 0.0000008)	0.0011	0.012			0.0072	0.0044		0.029	This publication
	Sunflower oil, 20 °C, 3 d	ND (< 0.000001)	0.0039	0.0036	0.0242			0.0142	0.0106		0.0643	
	Sunflower oil, 20 °C, 10 d	ND (< 0.000001)	ND (< 0.0000008)	0.0074	0.052	Nsa	Nsa	0.051	0.052		0.141	
	Sunflower oil, 40 °C, 2 h	ND (< 0.000001)	ND (< 0.0000008)	ND (< 0.000001)	0.0098		0.0064	0.0172	0.0038		0.0372	
	Sunflower oil, 70 °C, 2 h	ND (< 0.000001)–0.0032	ND (< 0.0000008)–0.005	ND (< 0.000001)–0.003	ND (< 0.0000008)–0.029		ND (< 0.0000008)–0.0451	0.00049–0.116	ND (< 0.0000007)–0.108		0.0012–0.338	
	Sunflower oil, 90 °C, 50 min	ND (< 0.000001)	0.0037	ND (< 0.000001)	0.0039		0.0019	0.0023	0.0020		0.014	
HIPS	Sunflower oil, 5 °C, 2 d	ND (< 0.000001)	ND (< 0.0000008)	ND (< 0.000001)	ND (< 0.0000008)		0.0095	0.0031	0.0051		0.0176	
	Sunflower oil, 20 °C, 0.5 h	ND (< 0.000001)	ND (< 0.0000008)	ND (< 0.000001)	ND (< 0.0000008)		0.0015	0.001	0.003		0.0054	
	Sunflower oil, 20 °C, 2 h	ND (< 0.000001)	ND (< 0.0000008)	ND (< 0.000001)	ND (< 0.0000008)		ND (< 0.0000008)	ND (< 0.0000009)	ND (< 0.0000007)		ND (< 0.0000008)	
	Sunflower oil, 40 °C, 2 h	ND (< 0.000001)	ND (< 0.0000008)	ND (< 0.000001)–0.003	ND (< 0.0000008)–0.0083		ND (< 0.0000008)–0.0085	ND (< 0.0000009)–0.0139	ND (< 0.0000007)–0.0053		ND (< 0.0000008)–0.0306	
	Sunflower oil, 40 °C, 24 h	ND (< 0.000001)	ND (< 0.0000008)	ND (< 0.000001)–0.0177	ND (< 0.0000008)–0.0728		0.0005–0.0911	0.0021–0.156	0.0008–0.0522		0.0017–0.248	
	Sunflower oil, 40 °C, 10 d	ND (< 0.000001)	ND (< 0.0000008)	ND (< 0.000001)–0.0046	ND (< 0.0000008)		ND (< 0.0000008)–0.0372	ND (< 0.0000009)–0.0036	ND (< 0.0000007)–0.0026		ND (< 0.0000008)–0.05	
	Sunflower oil, 70 °C, 2 h	ND (< 0.000001)–0.0014	ND (< 0.0000008)–0.0022	ND (< 0.000001)–0.018	ND (< 0.0000008)–0.019		ND (< 0.0000008)–0.0797	ND (< 0.0000009)–0.00386	ND (< 0.0000007)–0.087		ND (< 0.0000008)–0.244	
PS (dairy product)	10%, 40 °C, 10 d	–	< 0.0007	0.0122	< 0.0008	–	0.0121	< 0.0009	–	–	0.0243	Tsochatzis et al. (2020)
	50%, 40 °C, 10 d	–	0.0132	0.0289	0.0147	–	0.0694	0.0349	–	–	0.1611	
PS (yogurt)	10%, 40 °C, 10 d	–	< 0.0007	0.0142	< 0.0008	–	0.0142	< 0.001	–	–	0.0284	
PS (beverage)	10%, 70 °C, 2 h	–	< 0.0007	0.0098	< 0.0008	–	0.0112	< 0.0009	–	–	0.021	
	50%, 70 °C, 2 h	–	0.0128	0.0312	0.0122	–	0.0465	0.0289	–	–	0.1316	
OPP/alu (diary product)	10%, 40 °C, 10 d	–	< 0.0003	< 0.0003	–	–	< 0.0005	< 0.0005	–	–	–	
	50%, 40 °C, 10 d	–	< 0.0003	< 0.0003	–	–	< 0.0005	< 0.0005	–	–	–	
PA/PE (vegetables)	10%, 60 °C, 10 d	–	< 0.0004	< 0.0003	–	–	< 0.0005	< 0.0005	–	–	–	
PA/PP (vegetables)	10%, 60 °C, 10 d	–	< 0.0004	< 0.0003	–	–	< 0.0005	< 0.0005	–	–	–	
HIPS	20%, 40 °C, 10 d	0.0005	0.0005	Nsa	0.0005	Nsa	Nsa	Nsa	Nsa	0.003	0.0045	Gelbke et al. (2019)
GPPS	20%, 40 °C, 10 d	0.0005	0.0005	Nsa	0.001	Nsa	Nsa	Nsa	Nsa	0.011	0.012	
HIPS	50%, 40 °C, 10 d	< 0.011	Nd	0.3	Nd	Nsa	Nsa	Nsa	Nsa	0.6	0.9	
GPPS	50%, 40 °C, 10 d	< 0.011	Nd	0.034	Nd	Nsa	Nsa	Nsa	Nsa	0.2	0.234	
HIPS	50%, 40 °C, 10 d	0.0005	0.001	Nsa	0.022	Nsa	Nsa	Nsa	Nsa	0.141	0.165	
GPPS	50%, 40 °C, 10 d	0.0005	0.001	Nsa	0.013	Nsa	Nsa	Nsa	Nsa	0.125	0.14	
HIPS	95%, 60 °C, 10 d	0.01	Nd	1.4	Nd	Nsa	Nsa	Nsa	Nsa	1.0	2.41	
GPPS	95%, 60 °C, 10 d	< 0.011	Nd	0.332	Nd	Nsa	Nsa	Nsa	Nsa	0.4	0.732	
EPS	10%, 60 °C, 10 d	–	–	0.00608–0.00744	–	–	–	–	–	–	–	Song et al. (2019)
EPS recycled	10%, 60 °C, 10 d	–	–	< LOD	–	–	–	–	–	–	–	
EPS	3% acetic acid, 60 °C, 10 d	–	–	0.00305–0.00369	–	–	–	–	–	–	–	
EPS recycled	3% acetic acid, 60 °C, 10 d	–	–	< LOD –0.00246	–	–	–	–	–	–	–	
EPS	50%, 40 °C, 10 d	0.035–0.037	0.025–0.045	0.0077–0.02	0.017–0.022	–	0.0069–0.012	0.0054–0.0076	0.014–0.016	–	0.112–0.152^a^	Funke et al. (2018)
	Tenax, 40 °C, 10 d	ND (< 0.0000006)	ND (< 0.0000004)	0.0004–0.0054	ND (< 0.0000004)	–	0.0004–0.0091	0.0008–0.0052	0.0004–0.0031	–	0.0019–0.0228^a^	
	Sunflower oil, 40 °C, 10 d	ND (< 0.000015)–0.0042	ND (< 0.000001)	ND (< 0.000013)–0.127	ND (< 0.00001)	–	0.017–0.312	0.009–0.188	0.034–0.231	–	0.0022–0.858^a^	
	Sunflower oil, 40 °C, 24 h	ND (< 0.000015)–0.0026	ND (< 0.000001)–0.017	0.0007–0.023	ND (< 0.00001)–0.195	–	0.0025–0.158	0.0007–0.052	0.001–0.091	–	0.0059–0.485^a^	
HIPS	10%, 70 °C, 2 h	ND (< 0.0000012)	ND (< 0.0000008)	0.0006–0.002	ND (< 0.0000008) –	–	0.0006–0.0011	LOQ (< 0.0000035)–0.0009	0.001–0.0013	–	0.0029–0.0045^a^	
	10%, 20 °C, 2 h	ND (< 0.0000012)	ND (< 0.0000008)	ND (< 0.000001)–0.0011	ND (< 0.0000008)–0.0012–	–	ND (< 0.0000008)–0.0008	ND (< 0.0000009)–0.001	ND (< 0.0000007)–0.0013	–	ND (< 0.0000008)–0.0049^a^	
	50%, 70 °C, 2 h	ND (< 0.000001)–0.036	ND (< 0.0000008)–0.029	ND (< 0.000001)–0.012	0.0016–0.034	–	0.00005–0.022	0.00004–0.11	0.0001–0.018	–	0.014–0.144^a^	
	50%, 20 °C, 2 h	ND (< 0.000001)	ND (< 0.0000008)	ND (< 0.000001)–0.003	ND (< 0.0000008)–0.0025	–	0.00004–0.0054	0.00003–0.002	0.00008–0.0027	–	0.0058—0.013^a^	
EPS cup	50% EtOH	–	ND	ND	0.002	Nsa	Nsa	Nsa	Nsa	ND	0.002	Hirano et al. (2001) and Yamada et al. (2000a)
	50% heptane	–	0.0071	0.0013	0.0047	Nsa	Nsa	Nsa	Nsa	0.0019	0.015	
PS/HIPS/PSP cup	50% EtOH	–	0.0011	ND	0.0018	Nsa	Nsa	Nsa	Nsa	0.0041	0.007	
	50% heptane	–	ND	ND	0.0207	Nsa	Nsa	Nsa	Nsa	0.0426	0.0633	
Instant Food cup	*n*-Heptane, rt, 1 h	ND–0.001	ND–0.080	ND–0.145	0.001–0.749	ND–0.463	ND–0.491	ND–0.472	ND–0.752	–	0.002–2.609	Kaneko et al. (1999)
Dairy products cup	*n*-Heptane, rt, 1 h	ND–0.124	ND–0.475	ND–0.626	0.006–2.810	0.001–6.240	0.006–3.000	0.003–1.300	0.003–2.100	–	0.021–16.675	
Case for business use	*n*-Heptane, rt, 1 h	ND–0.013	ND–0.182	ND–0.301	0.009–1.580	0.006–0.767	0.011–2.170	0.004–0.888	0.006–1.450	–	0.043–7.338	
Disposable ware	*n*-Heptane, rt, 1 h	ND–0.001	ND–0.008	ND–0.007	0.004–0.047	0.002–0.040	ND–0.055	0.004–0.030	0.005–0.049	–	0.026–0.215	
Table ware	*n*-Heptane, rt, 1 h	ND–0.001	ND–0.009	ND–0.004	0.003–0.015	0.001–0.014	0–0.020	ND–0.008	ND–0.011	–	0.016–0.064	
All samples	Water, 60 °C and 95 °C, 0.5 h	ND	ND	ND	ND	ND	ND	ND	ND	–	–	
	20% EtOH, 60 °C, 0.5 h	ND	ND	ND	ND—0.003					–	–	
GPPS food case	Water, 60 °C, 0.5 h	ND	ND	ND	ND	ND	ND		ND	ND	ND	Kawamura et al. (1998a)
	20% EtOH, 60 °C, 0.5 h	ND	ND	ND	0.006^b^	ND	ND		ND	ND	0.006^b^	
	50% EtOH, 60 °C, 0.5 h	ND	ND	ND	0.036^b^	ND	ND		ND	ND	0.036^b^	
	*n*-Heptane, 25 °C, 1 h	ND	ND	ND	0.096^b^	0.024^b^	0.108^b^		ND	ND	0.228^b^	
HIPS cup	Water, 60 °C, 0.5 h	ND	ND	ND	ND	ND	ND		ND	ND	ND	
	20% EtOH, 60 °C, 0.5 h	ND	ND	ND	0.006^b^	ND	ND		ND	ND	0.006^b^	
	50% EtOH, 60 °C, 0.5 h	ND	ND	ND	0.024^b^	0.024^b^	0.036^b^		ND	ND	0.084^b^	
	*n*-Heptane, 25 °C, 1 h	0.012^b^	0.114^b^	0.36^b^	3.9^b^	5.7^b^	11.34^b^		3.24^b^	20.28^b^	24.67^b^	
PS foam bowl (donburi)	Water, 60 °C, 0.5 h	ND	ND	ND	ND	ND	ND		ND	ND	ND	
	20% EtOH, 60 °C, 0.5 h	ND	ND	ND	0.012^b^	ND	ND		ND	ND	0.012^b^	
	50% EtOH, 60 °C, 0.5 h	ND	ND	ND	0.042^b^	ND	ND		ND	ND	0.042^b^	
	*n*-Heptane, 25 °C, 1 h	ND	0.072^b^	0.084^b^	1.5^b^	0.48^b^	1.26^b^		0.42^b^	2.16^b^	3.816^b^	
EPS Chinese noodles	Water, 60 °C, 0.5 h	–	ND	ND	ND	ND	ND		ND	ND	ND	Kawamura et al. (1998b)
	20% EtOH, 60 °C, 0.5 h	–	ND	ND	ND	ND	ND		ND	ND	ND	
	50% EtOH, 60 °C, 0.5 h	–	ND	ND	ND	ND	ND		ND	ND	ND	
	*n*-Heptane, 25 °C, 1 h	–	ND	ND	0.0046	ND	0.0043		ND	0.0043	0.0089	
HIPS/PSP Chinese noodles	Water, 60 °C, 0.5 h	–	ND	ND	ND	ND	ND		ND	ND	ND	
	20% EtOH, 60 °C, 0.5 h	–	ND	ND	ND	ND	ND		ND	ND	ND	
	50% EtOH, 60 °C, 0.5 h	–	ND	ND	ND	ND–0.001	0.0013–0.0031		ND– 0.001	0.0013–0.0051	0.0013–0.0051	
	*n*-Heptane, 25 °C, 1 h	–	0.0051–0.0064	0.0023–0.0032	0.0447–0.0696	0.0268–0.0719	0.0589–0.1649		0.0393–0.0772	0.125–0.314	0.1771–0.3932	
HIPS Chow mein	Water, 60 °C, 0.5 h	–	ND	ND	ND	ND	ND		ND	ND	ND	
	20% EtOH, 60 °C, 0.5 h	–	ND	ND	ND	ND	ND		ND	ND	ND	
	50% EtOH, 60 °C, 0.5 h	–	ND	ND	0.001	0.0027	0.0079		0.0041	0.0147	0.0157	
	*n*-Heptane, 25 °C, 1 h	–	0.0224	0.1086	0.4483	0.3879	0.8621		0.4569	1.7069	2.2862	
HIPS/PSP Spaghetti	Water, 60 °C, 0.5 h	–	ND	ND	ND	ND	ND		ND	ND	ND	
	20% EtOH, 60 °C, 0.5 h	–	ND	ND	ND	ND	ND		ND	ND	ND	
	50% EtOH, 60 °C, 0.5 h	–	ND	ND	ND	ND	ND		ND	ND	ND	
	*n*-Heptane, 25 °C, 1 h	–	0.0029	0.0076	0.0968	0.0839	0.1774		0.0323	0.2936	0.4009	

*EPS* expanded PS, *GPPS* general purpose PS, *HIPS* high impact PS, *LOD* limit of detection, *Nd* no data and not specifically analysed, *ND* not detected, *Nsa* isomers not specifically analysed, – not reported, *OPP* oriented polypropylene, *PA* polyamide, *PE* polyethylene, *PP* polypropylene, *PS* polystyrene, *PSP* PS paper, *rt* room temperature

^a^Not in the publication. After checking the raw data by the CVUA-MEL, the results were supplemented by the sum of the oligomers

^b^Results originally given in µg/cm^2^, values presented here for better comparability calculated to mg/kg using a surface to mass ratio of 6 dm^2^/kg

**Table 3:** Contents (in mg/kg) of styrene dimers (SD) and styrene trimers (ST) in packaged food items

Food item	Material	Dimers			Trimers					Sum		References
		SD1	SD3	SD4	ST1	ST2	ST3	ST4	ST5	ST2, ST3, ST4, ST5	All Oligomers	
Crème fraiche	HIPS	ND (< 0.000015)	ND (< 0.00001)	ND (< 0.000013)	ND (< 0.00001)		ND (< 0.00001)–0.017	ND (< 0.000011)	ND (< 0.000008)		ND (< 0.000013)–0.017^a^	This publication
Coffee cream	HIPS	ND (< 0.000015)	ND (< 0.00001)	0.0012–0.0042	ND (< 0.00001)–0.0065		0.0024–0.076				0.036–0.123	
French fries	EPS menu box	ND (< 0.000015)	ND (< 0.00001)	0.0067–0.0076	ND (< 0.00001)		0.014–0.025	0.0078–0.014	0.028–0.037		0.057–0.084^a^	Funke et al. (2018)
Donut	EPS menu box	ND (< 0.000015)	ND (< 0.00001)	0.001	ND (< 0.00001)		0.0032	0.002	0.0084		0.015^a^	
Chop suey with chicken and rice	EPS menu box	ND (< 0.000015)	ND (< 0.00001)	0.0014	ND (< 0.00001)		0.0066	0.011	0.011		0.03^a^	
Leaf salad with vinaigrette	EPS menu box	ND (< 0.000015)	ND (< 0.00001)	0.0008	ND (< 0.00001)		0.0025	0.0014	0.0055		0.01^a^	
Cheese	EPS trailer	ND (< 0.000015)	ND (< 0.00001)	ND (< 0.000013)	ND (< 0.00001)–0.0059		ND (< 0.00001)–0.0088	ND (< 0.00001)–0.005	ND (< 0.00001)–0.0041		ND (< 0.000013)–0.024^a^	
Cooked ham	EPS trailer	ND (< 0.000015)	ND (< 0.00001)	ND (< 0.000013)	ND (< 0.00001)		ND (< 0.00001)	ND (< 0.000011)	ND (< 0.000008)		ND (< 0.000013)^a^	
Smoked Salmon	EPS trailer	ND (< 0.000015)	ND (< 0.00001)	ND (< 0.000013)	ND (< 0.00001)		ND (< 0.00001)	ND (< 0.000011)	ND (< 0.000008)		ND (< 0.000013)^a^	
Frozen cakes	EPS insulation material	ND (< 0.000015)	ND (< 0.00001)	0.0003–0.0008	ND (< 0.00001)		0.0002–0.0004	0.0001–0.0002	0.0007–0.0009		0.0014–0.0023^a^	
honey	EPS beehives	ND (< 0.000015)	ND (< 0.00001)	ND (< 0.000013)	ND (< 0.00001)		ND (< 0.00001)–0.0012	0.0002–0.0013	ND (< 0.000008)–0.0008		0.00019–0.0033^a^	
Yoghurt	HIPS yoghurt pots	ND (< 0.000015)	ND (< 0.00001)	ND (< 0.000013)	ND (< 0.00001)		ND (< 0.00001)	ND (< 0.000011)	ND (< 0.000008)		ND (< 0.000013)^a^	
Chocolate	HIPS CD-cover	0.00037	ND (< 0.00001)	0.016	0.3		0.106	0.203	0.058		0.707^a^	
Raw chicken	–	< 0.005	–	–	–	–	–	–	–	–	–	Genualdi et al. (2014)
yoghurt	–	< 0.005	–	–	–	–	–	–	–	–	–	
chocolate candy	–	< 0.005	–	–	–	–	–	–	–	–	–	
Noodle and soup	EPS	–	0.0007–0.0012	ND	0.0006–0.0015	Nsa	Nsa	Nsa	Nsa	0.0012–0.0021	0.0034–0.0039	Yamada et al. (2000b)
	HIPS/PSP	–	0.0004–0.0013	0.0003–0.0013	0.0015–0.0049	Nsa	Nsa	Nsa	Nsa	0.0038–0.0247	0.006–0.0298	
	HIPS/PSP improved container	–	ND–0.0015	ND–0.0002	0.0008–0.003	Nsa	Nsa	Nsa	Nsa	0.0018–0.0047	0.0026–0.0076	
	OPS/HIPS/PSP	–	ND–0.0007	ND–0.0002	0.0012–0.0016	Nsa	Nsa	Nsa	Nsa	0.0042–0.0069	0.0059–0.0093	
	OPS/HIPS/PSP improved container	–	ND–0.0003	ND	0.0004–0.001	Nsa	Nsa	Nsa	Nsa	0.0013–0.0019	0.0019–0.0026	
Only Noodle	PSP/HIPS	–	ND	ND–0.0004	0.0017	Nsa	Nsa	Nsa	Nsa	0.0113–0.0117	0.0138	
	PSP/HIPS improved container	–	ND	ND	0.0006	Nsa	Nsa	Nsa	Nsa	0.002	0.0026	
	OPS/HIPS/PSP	–	ND	ND	ND	Nsa	Nsa	Nsa	Nsa	0.0013	0.0013	
	OPS/HIPS/PSP improved container	–	ND	ND	ND	Nsa	Nsa	Nsa	Nsa	0.0016	0.0004	
Noodles & spaghetti	EPS	–	ND	ND	ND	ND	ND		ND	ND	ND	Kawamura et al. (1998b)
	HIPS	–	ND	ND	ND	ND	ND		ND	ND	ND	
	HIPS/PSP	–	ND	ND	ND–0.0156	ND–0.0154	ND–0.0254		ND–0.0095	ND–0.0503	ND–0.0624	
	HIPS/PSP/HIPS	–	ND	ND	ND	0.0093	0.0187		ND	ND–0.0369	ND–0.0423	
Soups	EPS	–	ND	ND	ND	ND	ND		ND	ND	ND	
rice	PSP	–	ND	ND	ND	ND	0.0076		ND	0.0076	0.0076	
	HIPS/PSP	–	ND	ND	0.0192	0.0094	0.0142		ND	0.0236	0.0428	
	PSP/HIPS	–	ND	ND	0.0057	ND	0.0076		ND	ND	0.0057	

*EPS* expanded PS, *HIPS* high impact PS, *Nsa* isomers not specifically analysed, *ND* not detected, – not reported, *OPS* oriented polystyrene, *PSP* polystyrene paper

^a^Not in the publication. After checking the raw data by the CVUA-MEL, the results were supplemented by the sum of the oligomers

**Table 4:** Contents (in mg/kg) of styrene dimers (SD) and styrene trimers (ST) in food contact materials

Food contact material	Food/simulant	Dimers			Trimers					Sum		References
		SD1	SD3	SD4	ST1	ST2	ST3	ST4	ST5	ST2, ST3, ST4, ST5	All Oligomers	
EPS	Ethyl acetate	5.36–6.93	114.7–123.9	13.06–13.69	153.5–370.7	4.08–7.62	34.7–56.3	11.02–17.98	14.29–24.29	–	352.3–743.8	Genualdi et al. (2014)
XPS	Ethyl acetate	10.77–15.00	140.0–169.2	26.92–54.23	669.2–1004	91.15–234.6	346.2–984.2	131.5–309.2	193.5–567.3	–	1608.7–3337.5	
HIPS	Ethyl acetate	6.07–40.60	< LOQ–2045	21.97–827.4	67.54–5652	255.7–1043	155.8–4841	702.1–1557	370.7–2775	–	2672.6–16,353.8	
GPPS	Ethyl acetate	30.8	578.4	200.0	1601	524.4	2343	800.4	1182	4849.8	7260.2	
ABS	Ethyl acetate	8.916	119.9	259.1	284.2	25.41	109.7	51.09	67.54	253.74	9832.94	
Food grade non-expanded PS	*n*-Heptane, 72 h, 10, 24 or 40 °C	–	–	–	–	–	–	–	–	4756 (all ST, complete extraction)	5402 (complete extraction)	Choi et al. (2005)
EPS	Cyclohexane/2-PrOH = 1/1	–	160	10	160	Nsa	Nsa	Nsa	Nsa	60	390	Hirano et al. (2001) and Yamada et al. (2000a, b)
PSP	Cyclohexane/2-PrOH = 1/1	–	150	50	980	Nsa	Nsa	Nsa	Nsa	2450	3630	
HIPS	Cyclohexane/2-PrOH = 1/1	–	20	10	540	Nsa	Nsa	Nsa	Nsa	3430	4000	
HIPS(lid)/PP (lunch box)	Vegetable oil	–	47.3/ND	–	260.0/ND	310.0/ND	960.0/ND	410.0/ND	690.0/ND	–	2677.3/ND	Sakamoto et al. (2000)
GPPS (lid)/PP (lunch box)	Vegetable oil	–	74.0–220/ND	–	670–1110/ND	370–630/ND	1130–1880/ND	480–880/ND	820–1310/ND	–	3700–5749.3/ND	
GPPS (lid)/HIPS (lunch box)	Vegetable oil	–	67.5/24.8	–	520/260	830/260	2270/590	1010/250	1770/500	–	6467.5/1884.8	
PSP(lid)/PSP (lunch box)	Vegetable oil	–	74.0/110.0	–	990/790	770/530	2150/1560	940/700	1620/1140	–	6744/4830	
Instant food cup	–	ND–30	40–250	10–350	160–1610	ND–2320	ND–1660	ND–840	10–1440	–	340–6580	Kaneko et al. (1999)
Dairy products cup	–	ND–20	ND–280	ND–170	40–1680	10–1210	30–1150	10–460	30–870	–	120–4620	
Case for business use	–	ND–30	60–270	80–320	410–1240	270–730	630–1640	310–670	450–1240	–	2240–5870	
Disposable ware	–	10–40	70–340	200–320	860–1690	310–1660	ND–1850	370–770	690–1300	–	4397–6060	
Table ware	–	ND–30	70–480	40–880	500–2090	90–2830	ND–1660	110–990	200–1800	–	1480–6780	
GPPS	Cyclohexane/2–PrOH = 1/1	ND–40	40–430	30–220	470–3560	190–3720	510–7380		180–2000	880–13,100	1540–17,160	Kawamura et al. (1998a)
HIPS	Cyclohexane/2–PrOH = 1/1	ND–60	30–600	ND–860	1300–5600	1600–2330	3400–8000		920–2000	5920–14,000	7520–20,260	
PS foam	Cyclohexane/2–PrOH = 1/1	ND–60	40–450	20–380	340–3770	70–3800	130–7900		ND–2200	210–13,780	760–18,170	
EPS	–	–	50–100	10–30	300–450	30–90	40–180		20–70	90–340	430–760	Kawamura et al. (1998b)
PSP	–	–	60	170	1480	1200	2660		1030	4890	6600	
HIPS	–	–	70	310	1800	2350	4900		1920	9170	11,350	
HIPS/PSP	–	–	60–190	90–250	860–2130	740–3050	1780–7350		670–2250	3220–12,650	4820–15,220	
PSP/HIPS	–	–	70	180	1530	840	2130		780	3750	5530	
HIPS/PSP/HIPS	–	–	70–130	100–210	960–1420	880–2300	4740		1900	4640–8940	5780–10,230	

*LOQ* limit of quantification, *ND* not detected, *Nsa* isomers not specifically analysed, – not reported

### Chemical inputs

Structural information is needed to perform in silico prediction. The chemical structures, the CAS registry numbers or the SMILES (Simplified Molecular Input Line Entry System) of SO were used as the entry/input information for the in silico tools. SMILES were obtained by ACD/ChemSketch, while CAS registry numbers were obtained from Scifinder^®^ (https://scifinder.cas.org). Isomeric SMILES were used for isomeric compounds.

### Computer tools

The QSAR Toolbox (https://www.oecd.org/chemicalsafety/risk-assessment/oecd-qsar-toolbox.htm) is an expert tool developed by OECD in close collaboration with ECHA. It consists of a free software application able to profile and group chemicals, retrieve experimental data and fill the gaps by read across, trend analysis or (Q)SARs. The OECD QSAR Toolbox version 4.4 released in February 2020 was used for all investigations. For each styrene oligomer the endpoint genetic toxicity was selected. Profiling (in silico estimation) for genotoxicity was done, applying the following methods: DNA and protein binding by OASIS, DNA and protein binding by OECD for the General Mechanistic category, as well as DNA alerts for AMES, Chromosomal Aberration and Micronucleus Test by OASIS, in vitro mutagenicity (Ames test) alerts by ISS, in vivo mutagenicity (Micronucleus) alerts by ISS and Protein binding alerts for Chromosomal aberration by OASIS for the Endpoint specific category. Detailed information and references for the underlying mechanistic rules and training sets are provided within the QSAR Toolbox. Performance was recently evaluated by Pradeep et al. (2021).

In addition, read-across analysis was performed using a category approach, and for data profiling and gathering the following databases were used according to the selected endpoint: ECHA CHEM, Bacterial mutagenicity ISSSTY, Genotoxicity and Carcinogenicity ECVAM, Genotoxicity OASIS, Genotoxicity pesticide EFSA, Micronucleus ISSMIC, Micronucleus OASIS, Toxicity Japan MHLW and Transgenic rodent database. Categorization was achieved by successively applying the following search criteria: structure similarity (≥ 40%), DNA binding by OECD (Michael addition >> P450 Mediated Activation to Quinones and Quinone-type Chemicals >> Arenes) and Chemical elements (Group 14—Carbon C). Since for some SO after these categorisation steps still many substances remained, we increased the inclusion limit for structural similarity to ≥ 50%. Classification of the SO and analogues as positive/negative with regard to genotoxicity was performed as read-across prediction for “genetic toxicity” taking into account all studies with the endpoints “gene mutation, in vitro cytogenicity/chromosome aberration study in mammalian cells, in vitro damage and/or repair study, in vitro gene mutation study in mammalian cells and in vivo mammalian somatic cell study: cytogenicity/erythrocyte micronucleus”. Log K_OW_ was used for choosing the most similar analogues. The results were accepted as provided by the QSAR toolbox, irrespective of the actual quality of the underlying studies (e.g. GLP or OECD TG compliance), which cannot be reliably assessed from the Toolbox.

DEREK Nexus is a commercial knowledge-based software developed by Lhasa Ltd. (2022a) (Nexus version 2.2.2) (Marchant et al. 2008). The test substance is structurally classified by the tool, and the recognised features are compared with a specific reference set of structural features and respective classification rules based on genotoxicity test data. DEREK version 6.0.1 was used for the analysis applying the knowledge base “Derek KB 2018 1.1”. The software predicts the potential for gene mutation in bacteria (Ames test). In addition, rule-based alerts for chromosome damage in vitro*/*in vivo, mutagenicity in mammalian cells in vitro, mutagenicity in vivo, non-specific genotoxicity in vitro*/*in vivo, photo-induced chromosome damage in vitro, photo-induced non-specific genotoxicity in vitro*/*in vivo and photomutagenicity in vitro are provided whose predictivity for effects seen in chromosomal aberration assays in vitro is acceptable (Foster 2021)*.* The reasoning level for which results/alerts were supposed to be shown was set at “at least equivocal”. Derek Nexus contains alerts for these multiple endpoints. The version used in this work contains 132 active alerts for bacterial mutagenicity, together with reasoning rules and secondary functionality that evaluates potentially misclassified and unclassified features in compounds that do not activate bacterial mutagenicity alerts or examples. For bacterial in vitro mutagenicity DEREK model is primarily based on data from the Ames test conducted following the standard test protocol (OECD TG 471). If activity is observed in a non-standard assay or protocol, this is mentioned in the comments. The Lhasa Ames Test Reference Set is the database of reference and is not publicly available. It is composed of several curated Ames datasets comprising 12,196 compounds (5813 positive and 6383 negative). This test reference is not a training set per se*,* but it is composed by illustrative examples. Performance data are presented by the company and reported in the literature (Sutter et al. 2013; Jolly et al. 2015; Williams et al. 2016; Hemingway et al. 2017; Slavov et al. 2018; Morita et al. 2019; Tennant et al. 2019).

Sarah Nexus is a commercial statistics-based software developed by Lhasa Ltd. (2022b) (Nexus version 2.2.2). Sarah version 3.0.0 was used applying the model “Sarah Model—2.0” for the endpoint “mutagenicity in vitro”, predicting chemicals to be mutagenic or non-mutagenic in bacteria (Ames test). The model is based primarily on data from bacterial reverse mutation assays without defining a specific experimental protocol (e.g. according to OECD TG 471). However, strain profiles have been implemented into Sarah Nexus to aid the expert review of supporting Ames strain data for both hypotheses and individual structures. The training set for Sarah Model 2.0 contains 11,774 individual structures (updated to June 2020) comprising 5780 mutagens and 5994 non-mutagens. It is not entirely publically available due to the proprietary nature of the model. Statistic metrics are provided along with the model in the software as well as in the literature (Barber et al. 2016; Hemingway et al. 2017; Slavov et al. 2018). Test substances are structurally classified according to their functional groups, and similar substances are searched in the training set to generate a prediction. For each prediction the model calculates a confidence score based on the similarity of the substances found and the confidence of the genotoxicity results from the training set. Generally, the equivocal borderline and sensitivity level were set at 8%, and the reasoning type was set as “weighted”.

The new Danish (Q)SAR Database is a freely available database containing model estimates for more than 600,000 substances. It has been developed by the DTU National Food Institute (Division of Diet, Disease Prevention and Toxicology, Technical University of Denmark, http://qsar.food.dtu.dk) in cooperation with the Danish Environmental Protection Agency, the Nordic Council of Ministers and the ECHA. The models for Bacterial Reverse Mutation Test (Ames) and for the chromosomal aberrations in CHL (Chinese hamster lung) fibroblasts were chosen for the assessment. Ames test data used were restricted to standard Ames test of Salmonella Typhimurium strains TA98, TA100, TA1535 and either TA1537 or TA97. Strains TA102 and TA1538 were also selected in case of equivocal results of other strains. Tests were only considered if performed according to the experimental protocol described in OECD TG 471 (1997). The statistic metrics for the model, obtained by leave‐many‐out cross‐validation, are reported as follows: sensitivity = 84.3%, specificity = 85.7%, concordance = 84.9%. For the chromosomal aberrations endpoint, data were taken mainly from a single source, the “Data Book of Chromosomal Aberration Test In Vitro” and generated using similar experimental protocols to that described in OECD TG 473 (1997). Statistics obtained by leave‐many‐out cross‐validation are reported as follows: sensitivity = 74.6%, specificity = 75.2%, concordance = 74.9%.

Discrimination between the enantiomers/diastereomers ST2–ST5 was not possible for all in silico tools. Hence, these substances were treated as one substance.

## Results

### Contents of SO in FCM and migration into food/food simulants

Data presented in Tables 2, 3 and 4 were collected from publicly available literature and own experiments. The tables summarize contents of the investigated SO measured in food or food simulants as well as in different FCM from PS.

As already pointed out by Gelbke et al. (2019), many studies were performed reporting migration of SO from food containers. A majority of them are Japanese studies dealing with popular Japanese or Asiatic food, such as noodle soups or instant food (Table 3) (Kawamura et al. 1998b, 1998c), warmed up and stored under conditions that do not reflect the common Western usage of PS food packaging. In course of the analysis of SD and ST in food typical packaged in PS in Europe, like yogurt, bakery foodstuff and some raw meat containers, no oligomers were detected (Genualdi et al. 2014).

When using food simulants (Table 2), in some cases n-heptane was used (Hirano et al. 2001; Kaneko et al. 1999; Kawamura et al. 1998a, 1998b; Yamada et al. 2000a), which is not a food simulant according to Commission Regulation (EU) No 10/2011 and thus might not lead to results comparable to real food. Furthermore, the measured content of the SD and ST in food simulants is quite high when typical simulants for fatty food are used. Specifically, using of 95% (vol%) ethanol (EtOH), *n*-heptane or 50% (vol%) EtOH leads to significantly higher levels of SO in the simulants when compared to real foods they represent (compare Table 3). Hence, the results with these food simulants should be treated with caution. Nevertheless, data show that SD and ST are present in PS materials in contact with food (Table 4) and are transferred to real foodstuff in significant amounts (Table 3).

We investigated the migration of SO into the food “sunflower oil” under different conditions (Table 2) as well as the SO contents in crème fraiche and coffee cream, being typical fatty foods stored in PS at low temperatures (Table 2). The migration into sunflower oil increases with increasing exposure time and temperature. Migration of up to 0.388 mg/kg sunflower oil was measured at 70 °C/2 h. However, already at low temperatures, a significant migration of SO was detectable: the overall migration of SO into sunflower oil at 5 °C/3 days, 20 °C/0.5 h or 20 °C/2 h, being a typical application of a vinaigrette in a “salad-to-go” product, was found at levels of up to 0.0643 mg/kg oil. Storage of fatty foods in PS packaging (HIPS) for longer times resulted in comparable contents of SO of up to 0.017 mg/kg créme fraiche and up to 0.123 mg/kg coffee cream (Table 3). The main oligomers detected in food were SD4 and ST.

### In silico genotoxicity prediction

To predict genetic toxicity of SO, two knowledge-based (expert rule) and two statistics-based models were applied. The summary of the in silico assessment is presented in Table 6.

#### OECD (Q)SAR toolbox

All genotoxicity prediction profilers provided by the OECD (Q)SAR toolbox gave the result “no alert found” (see Table S1 in the supplementary material)—with one exception: for all SD and ST the profiler “DNA binding by OECD” resulted in the following alert: “Michael addition >> P450 Mediated Activation to Quinones and Quinone-type Chemicals >> Arenes”. However, this is a misclassification, since for these types of substances, hydroxylation of the aromatic ring or the alkylic side chain followed by Phase-II-conjugation can be expected. Similarly as seen, e.g., for the substance triphenylmethane (CAS 519-73-3) (Cornish et al. 1964). Instead, formation of quinones is unlikely. In addition, analogues for data gap filling through read across were identified in accordance with the procedure described above. Considering that SD and ST have very similar structures, the analogues found using the chosen grouping criteria are nearly the same for all the target SO (see Table 5). In fact, almost all of them are linear alkanes branched with two or three aryl groups. For all analogues plotted in the final read-across graph, the available genotoxicity studies (mainly bacterial reverse mutation and chromosomal aberration tests) were negative; hence, all SD and ST were classified as non-genotoxic by the OECD (Q)SAR toolbox (see Table S2 for detailed reports of the results).

**Table 5:**
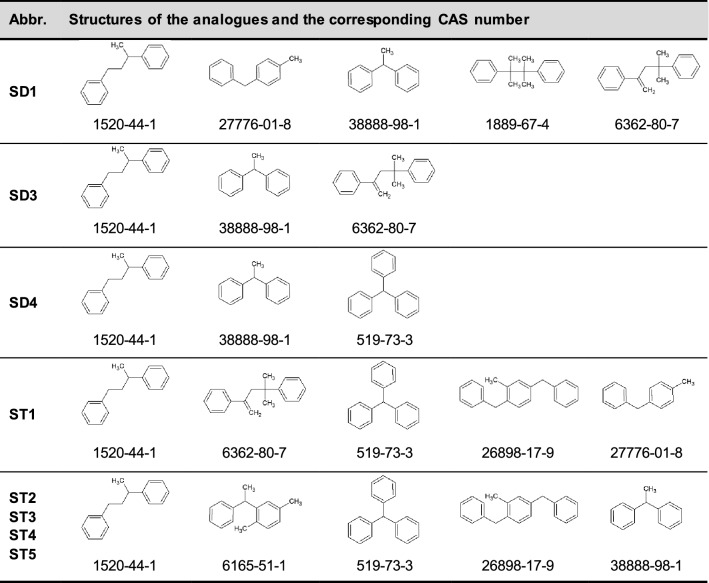
Analogues of styrene dimers (SD) and styrene trimers (ST) identified by OECD (Q)SAR Toolbox and selected for read-across analysis

#### DEREK/Sarah Nexus

The second knowledge-based software used was the commercial DEREK Nexus (Lhasa Ltd. 2022a). The chemical structures of the SO were compared with a specific reference set of structural features and respective classification rules based on genotoxicity test data. With respect to bacterial mutagenicity, the software classified all SO tested as “inactive with no misclassified or unclassified features”. Also, no alerts were found at the selected reasoning level for chromosomal damage in vitro and in vivo as well as all other genotoxicity endpoint selected (see methods section). Within ICH M7 workflow, DEREK Nexus is usually used in combination with the statistics-based software for mutagenicity prediction Sarah Nexus (Lhasa Ltd. 2022b). According to the Sarah model, input structures are split into relevant fragments that are further analysed through a machine-learning method. All SO were classified as negative (with respect to bacterial reverse mutation) with the only exception of SD4, which gave an equivocal result. For each prediction, confidence scores were assigned (Table S3—Supplementary materials). Prediction for SD1 showed the highest confidence value of 56%, indicating negative genotoxicity test results and high similarity for the selected substances from the reference database. For SD3 and ST1 the values were quite similar being at 40% and 37%, respectively, while the prediction for the trimeric isomers ST2, ST3, ST4 and ST5 came with a moderate/low confidence of 16% (equivocal borderline was set at 8%). However, this lower confidence value does not result from positive or equivocal genotoxicity test results in the reference database but from the comparatively lower similarity of the reference substances to the ST. Still, it can be concluded that there is some uncertainty at least in the negative result for the trimeric isomers.

#### Danish (Q)SAR Database

The Danish (Q)SAR Database consists of a repository of model estimates for more than 600,000 substances from free and commercial platforms. The selected models for genotoxicity were the in vitro Ames test and the chromosomal aberration test in Chinese Hamster Lung (CHL) fibroblasts. The predictions are given with an appropriate qualifier (NEG, POS, INC), probabilities for being positive and an information whether or not the structure is in the respective applicability domain (Table S4). All SO were predicted to be non-genotoxic (with respect to gene mutation and clastogenicity) and were inside the applicability domain (NEG_IN) with the only exception of SD4 for which the Ames model prediction was negative but out of the applicability domain (NEG_OUT), while the chromosomal aberration model prediction was inconclusive and out of the applicability domain (INC_OUT).

In contrast to the results from the OECD (Q)SAR Toolbox and DEREK Nexus, the genotoxicity predictions for SD4 from the statistics-based tool Sarah Nexus and the DTU database are not clearly negative but equivocal/inconclusive. However, a deeper investigation of the analogues found for SD4 by the QSAR Toolbox showed that neither of them includes a cyclobutane fragment (Table 5). For this reason, six cyclobutane derivatives were selected by expert judgment and used for further investigation (SD4-1 to SD4-6. Tables S3 and S4). To the best of our knowledge, no publicly available data on genotoxicity testing exists for any of these substances. Hence, they were also tested in silico. With respect to DEREK Nexus the chosen analogues were once again classified as “inactive with no misclassified or unclassified features”. Sarah Nexus prediction resulted in two equivocal outcomes for SD4-1 and SD4-2, while for the remaining analogues the outcome was negative with a confidence value of 26% for SD4-3, SD4-4, SD4-5 and 23% for SD4-6. The DTU chromosomal aberration model predicted all the SD4 analogues as inconclusive and out of the applicability domain (INC_OUT). The Ames model resulted in a negative prediction for genotoxicity inside the applicability domain (NEG_IN) for SD4-6, negative genotoxic prediction out of the applicability domain (NEG_OUT) for SD4-2, SD4-3 and SD4-5, and finally an inconclusive result out of the applicability domain (INC_OUT) for SD4-1 and SD4-4.

## Discussion

### Migration into food (simulants)

Overall, in most cases migration of SD is significantly lower than migration of ST, depending on the food simulants, temperature and time (Table 2). In comparison to the content measured in FCM, the migration of SO into foodstuffs or food simulants is very low. With respect to literature data, overall SD and ST content in real food and migration into simulants with up to 20% EtOH is below or around 50 µg/kg. In food simulants used to simulate foods with high fat content, such as 95% EtOH or n-heptane, overestimation of migration in comparison to real food is observed—especially if long contact time and high temperatures are used (Table 2). This might be due to the swelling of the PS material and subsequent “extraction”. Applying the food “sunflower oil” also can result in comparably high migration of SO (up to 0.338 mg/kg), especially for long migration times (up to 10 days) or high temperatures (up to 70 °C) (Table 2). For lower temperatures and contact times, e.g. 20 °C/2 h, the migration into sunflower oil was significantly lower. These conditions resemble real conditions for “salad to go” and migration is comparable to contents in a sample of “leaf salad with vinaigrette” containing most likely vegetable oil (0.01 mg/kg food, Table 3).

Migration conditions used should mirror the worst foreseeable conditions to include all possibilities in terms of simulants, time and temperature. Coffee cream and crème fraiche are two natural occurring examples that combine high surface/volume ratio and a fatty food matrix, which results in comparably high migration values (Table 3). In coffee cream, the sum of all oligomers was in the range of 0.036–0.123 mg/kg food. According to EFSAs “Note for guidance” (EFSA 2008), the proof of the absence of genotoxicity is necessary, but not sufficient to ensure safety of such comparably high migration values.

As amended by the directive 85/572/EEC, a correspondence between food and simulant is needed. Therefore, more data for real food, which in Europe is typically in contact with PS FCM (e.g. meat, yoghurt, vegetable oil like sun flower oil), as well as for the migration into simulants with respect to the corresponding conditions laid down in Regulation (EU) No 10/2011, are needed in order to clarify the suitability of simulants. The presented data indicate that optimization of migration conditions as well as food simulants used is possible in order to represent real life conditions. From the available data it seems likely that 95% EtOH or alkanes in combination with long migration times and/or elevated temperatures overestimate migration—probably due to the swelling of the PS material. The use of sunflower oil being a real food in combination with short incubation times and low temperatures might pave the road for realistic data, if analytical methods are available.

### In silico genotoxicity assessment

The present study was designed to predict the genotoxicity of SO as a part of a WoE approach applying in silico methodologies. Such methods, with respect to any traditional testing approaches, are less time consuming, more cost effective and can be used to screen and prioritize chemicals in hazard assessment. Although computational approaches have not yet gained full regulatory acceptance, their application is increasingly encouraged by many regulatory authorities as an integrated tool of an overall evidence framework. Because of the large and meaningful training datasets build on Ames assays, bacterial mutagenicity is one of the most modelled endpoint among all genetic endpoints.

In accordance with internationally recognized scientific principles of in silico modelling and corresponding guidelines (Benigni et al. 2019; ECHA 2008; Frenzel et al. 2017), several tools should be used for in silico genotoxicity prediction, preferably including both, a knowledge- and a statistics-based model. In fact, combining these two complementary systems is crucial to perform a reliable computational toxicology assessment. In the present study, four independent software programs based on different prediction approaches were used for each styrene oligomer. The rule-based DEREK nexus and the statistics-based Sarah Nexus are currently used as complementary (Q)SAR methods to meet the ICH-M7 guidelines. The Danish DTU database, as statistics-based tool, was used to retrieve genotoxicity predictions, while the OECD (Q)SAR toolbox, as expert rule- based, was used to perform read-across analysis employing data from identified analogues.

With the exception of SD4, all SD and ST were estimated to be non-genotoxic (with respect to gene mutation and clastogenicity) by all in silico models (Table 6), though in some cases the levels of confidence were fairly low. For SD4 and some analogues, the statistics-based tools Sarah Nexus and DTU database overall gave equivocal or inconclusive results, sometimes out of the applicability domain.

**Table 6:** Short summary of the results of the in silico genotoxicity modelling/prediction for styrene dimers (SD) and styrene trimers (ST) using different tools

Styrene oligomer	OECD QSAR toolbox profiler	OECD QSAR toolbox read across	DEREK Nexus	SARAH Nexus	DTU QSAR Ames test	DTU QSAR chromosomal aberration
SD 1	No alert found	Negative	Inactive	Negative	Negative	Negative
SD 3	No alert found	Negative	Inactive	Negative	Negative	Negative
SD 4	No alert found	Negative	Inactive	Equivocal	Negative^a^	Inconclusive^a^
ST 1	No alert found	Negative	Inactive	Negative	Negative	Negative
ST 2–ST 5	No alert found	Negative	Inactive	Negative	Negative	Negative

^a^Substance is reported to be outside of the applicability domain

Through the OECD (Q)SAR toolbox read-across analysis, all SO are assessed to be negative with respect to the considered genetic endpoints (Table 6). The identification of suitable analogues using existing information derived from similar chemicals is necessarily expert driven.

Overall, the two knowledge-based and the two statistics-based models support each other’s outcome. DEREK Nexus confirms the results obtained with the OECD (Q)SAR toolbox and, for the statistics-based Sarah Nexus software as well as for the DTU tool, SD4 is the only oligomer with an inconclusive result. In addition, the concentration of SD4 in the test solution of the only publicly available in vitro study (Nakai et al. 2014) was very low. But migration of SD4 into food simulants and real food has been observed (Tables 2, 3). For instance, migration into coffee cream was 0.0012–0.0042 mg/kg food and higher than for any other SD (Table 3). Therefore, the remaining uncertainty about the genotoxic potential of SD4 should be reduced. According to the EFSA Note for Guidance (EFSA 2008), an Ames test and an in vitro micronucleus assay according to the respective OECD guidelines should be performed with SD4 (*trans*-1,2-diphenylcyclobutane) in order to exclude genotoxicity.

Specific migration data for each single ST2, ST3, ST4 and ST5 stereoisomers are available only for some of the investigations reported in the literature, and although isomeric chemical structures can be apparently sketched out, none of the in silico tools tested could actually discriminate between them. In fact, the used (Q)SAR models are built on bi-dimensional (2D) molecular descriptors; therefore, a clear conclusion on the genotoxicity concern using the computational approach is not possible for the specific isomers. Despite this, ST2, ST3, ST4 and ST5 were assessed to be non-mutagenic and non-clastogenic by all methods applied here.

With the exception of SD4, the performed in silico assessment gave new evidence and confidence in the conclusion that SD and ST are non-genotoxic (with respect to gene mutation and clastogenicity). For SD4 the absence of genotoxicity should be proven in order to reduce the uncertainty further. It has to be stressed that for all SD and ST the chosen analogues/databases were more or less the same, and the only existing in vitro study (Nakai et al. 2014) was also performed with a mixture of SO. Hence, for risk assessment the SD and ST should be rather treated as a group than as single substances. As a conclusion, with respect to the tiered approach laid down in the EFSA Note for Guidance (EFSA 2008), the overall migration of SD1, SD3, ST1, ST2, ST3, ST4 and ST5 into food of up to 50 µg/kg food does not raise any health concerns, based on the currently available in silico and in vitro data.

### Endocrine activity of SO

Several toxicological studies have investigated the potential activity of SO to act as endocrine disruptors (Date et al. 2002; Gelbke et al. 2018; Ohyama et al. 2007, 2001; Yanagiba et al. 2008), but the concerns that were derived from these studies have been always contentious. In 1998 the Environmental Agency of Japan (JEA 1998) has listed SO as “chemicals suspected of having endocrine disrupting effects”. In 2000, they were withdrawn from that list because the risk estimated was only low. In Europe, too, SO were included in the list of endocrine disruptors published by the EU and then withdrawn in 2002 (BKH 2002). Gelbke et al. (2018) performed a literature review on the potential role of SO as endocrine disruptors using a weight of evidence (WoE) approach. This comprehensive review concluded that “the strongest in vitro and in vivo screening studies including non-guideline investigations in experimental animals do not indicate an endocrine disruption of SO for estrogenic or androgenic axis. Although the data on potential interference with the thyroid are less clear, the lack of effects on thyroid weight and histopathology support the conclusion that SDT do not act as ED on this target. But according to the definition of EFSA (2013) and WHO/IPCS (2002) it cannot be excluded with any certainty that SDT may act as EAS”.

Therefore, it can be concluded that SO might exhibit some endocrine activity, but OECD guideline conform studies from several independent laboratories that prove or exclude this activity are still missing.

## Supplementary Information

Below is the link to the electronic supplementary material.

Supplementary file1 (PDF 1344 KB)

## Abbreviations

CHL: Chinese Hamster Lung fibroblasts
DEREK: Deductive Estimation of Risk from Existing Knowledge
ECHA: European Chemical Agency
EFSA: European Food Safety Authority
EtOH: Ethanol
OECD: Organization for Economic Co-operation and Development
(Q)SAR: (Quantitative) structure–activity relationship
PS: Polystyrene
