# Supplementary material for: Migration of styrene oligomers from food contact materials: in silico prediction of possible genotoxicity
Source: Arch Toxicol. 2022 Aug 13;96(11):3013–32. doi: 10.1007/s00204-022-03350-x (PMC9376037; doi:10.1007/s00204-022-03350-x)
Supplement: Supplementary file 1 — Supplementary file1 (PDF 1344 KB) [file 204_2022_3350_MOESM1_ESM.pdf]

Supplementary material to E. Beneventi et al., "Migration of styrene oligomers from food contact materials: in silico prediction of possible genotoxicity".

Table S 1: Results of OECD QSAR-toolbox genotoxicity profilers for styrene oligomers

Styrene oligomer	SD 1	SD 3	SD 4	ST 1	ST 2 - ST 5 (different isomers not shown)
**Substance identity**					
Structure	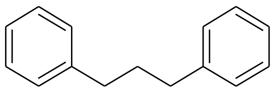	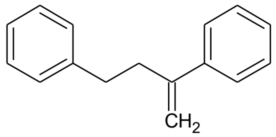	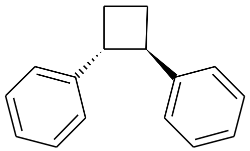	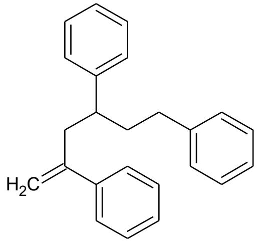	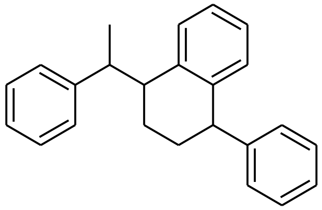
CAS number	1081-75-0	16606-47-6	20071-09-4	18964-53-9	26681-79-8
Chemical name	1,3-diphenylpropane	2,4-Diphenyl-1-butene	trans -1,2- diphenylcyclobutan	2,4,6- triphenyl-1-hexen	1a-phenyl-4a-(1-phenylethyl)-1,2,3,4 tetrahydronaphtale
SMILES	C(Cc1ccccc1)Cc1ccccc1	C=C(CCc1ccccc1)c1ccccc1	C1C[C@H](C1c1ccccc1)c1ccccc1	C=C(CC(CCc1ccccc1)c1ccccc1)c1ccccc1	CC(C1CCC(c2ccccc2)c2ccccc12)c1ccccc1
**Profilers**					
General Mechanistic					
Protein binding by OECD	No alert found	No alert found	No alert found	No alert found	No alert found
Protein binding by OASIS	No alert found	No alert found	No alert found	No alert found	No alert found
DNA binding by OASIS	No alert found	No alert found	No alert found	No alert found	No alert found
DNA binding by OECD	Michael addition >> P450 Mediated Activation to Quinones and Quinone-type Chemicals >> Arenes	Michael addition >> P450 Mediated Activation to Quinones and Quinone-type Chemicals >> Arenes	Michael addition >> P450 Mediated Activation to Quinones and Quinone-type Chemicals >> Arenes	Michael addition >> P450 Mediated Activation to Quinones and Quinone-type Chemicals >> Arenes	Michael addition >> P450 Mediated Activation to Quinones and Quinone-type Chemicals >> Arenes

Endpoint Specific					
DNA alerts for AMES, CA and MNT by OASIS	No alert found	No alert found	No alert found	No alert found	No alert found
Aquatic toxicity classification by ECOSAR	Neutral Organics	Neutral Organics	Neutral Organics	Neutral Organics	Neutral Organics
in vitro mutagenicity (Ames test) alerts by ISS	No alert found	No alert found	No alert found	No alert found	No alert found
Protein binding alerts for Chromosomal aberration by OASIS	No alert found	No alert found	No alert found	No alert found	No alert found
in vivo mutagenicity (Micronucleus) alerts by ISS	No alert found	No alert found	No alert found	No alert found	No alert found

Table S 2: Results of OECD QSAR-toolbox genotoxicity prediction via read-across to analogue substances; Notice to the reader: Additional tests for the different analogue substances may be available (in summary) in the respective registration dossier on the website of the European Chemicals Agency (ECHA). The OECD toolbox does not give information from the ECHA database in the output files, but still uses available information for read-across assessment. Output was taken as provided by the OECD toolbox and only corrected for obvious mistakes.

**Substance**	**SD 1**	Analogue #1	Analogue #2	Analogue #3	Analogue #4	Analogue #5
**Substance identity**						
Structure	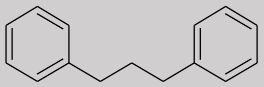	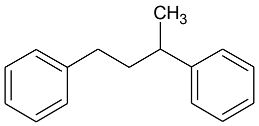	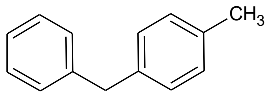	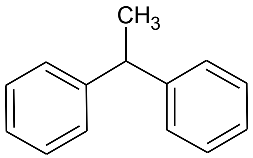	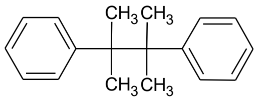	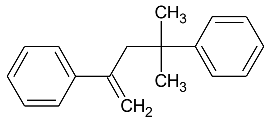
CAS number	1081-75-0	1520-44-1	27776-01-8	38888-98-1	1889-67-4	6362-80-7
Chemical name	1,3-diphenylpropane	1,3-diphenylbutane	Benzene, methyl(phenylmethyl)-	Diphenylethane	2,3-Dimethyl-2,3-diphenylbutane	2,4-Diphenyl-4-methylpentene-1
SMILES	C(Cc1ccccc1)Cc1ccccc1	CC(CCc1ccccc1)c1ccccc1	Cc1ccc(Cc2ccccc2)cc1	CC(c1ccccc1)c1ccccc1	CC(C)(c1ccccc1)C(C)(C)c1ccccc1	CC(C)(CC(=C)c1ccccc1)c1ccccc1
**Profiles used for grouping/subcategorization**						
Aryl (Organic functional groups) (primary grouping)	Aryl	Alkane, branched with tertiary carbon;<br>Aryl	Aryl;<br>Alkyl (hetero)arenes;<br>Alkyl-, alkenyl- and alkynyl (hetero)arenes	Aryl	Aryl	Alkene moiety ;<br>Allyl;<br>Aryl
DNA binding by OECD (subcategorization)	Michael addition >> P450 Mediated Activation to Quinones and Quinone-type Chemicals >> Arenes					
Chemical elements (subcategorization)	Group 14 - Carbon C					
Structure similarity (subcategorization)	[90%,100%]	[70%,80%)	[60%,70%)	[60%,70%)	[60%,70%)	[60%,70%)
**Predictions**						
Sublevel	Genetic Toxicity					

Endpoint	Gene mutation <OR> in vitro cytogenicity / chromosome aberration study in mammalian cells <OR> in vitro DNA damage and/or repair study <OR> in vitro gene mutation study in mammalian cells <OR> in vivo mammalian somatic cell study: cytogenicity / erythrocyte micronucleus					
Unit	Gene mutation I					
Reference	QSAR Toolbox Database: ECHA REACH <OR> Genotoxicity OASIS <OR> Toxicity Japan MHLW					
Prediction approach	Read-across					
Value	**negative**					
**Data used for prediction**						
Sublevel		Genetic Toxicity				
Endpoint		Gene Mutation				
Test description		Test organisms (species): Salmonella typhimurium<br>Endpoint: Gene mutation<br>Test type: Bacterial Reverse Mutation Assay (e.g. Ames Test)<br>Type of method: in Vitro<br>Strain: No Strain Info<br>Year: 1/1/2009 12:00:00 AM<br>Reference source: J. Chem. Inf. Model., 2009, 49 (9), pp 2077–2081<br>Author: Hansen K. et al.	Test organisms (species): Salmonella typhimurium<br>Endpoint: Gene mutation<br>Test type: Bacterial Reverse Mutation Assay (e.g. Ames Test)<br>Type of method: in Vitro<br>Strain: No Strain Info<br>Year: 1/1/2009 12:00:00 AM<br>Reference source: J. Chem. Inf. Model., 2009, 49 (9), pp 2077–2081<br>Author: Hansen K. et al.	Test organisms (species): Other Test organisms (species)<br>Endpoint: Gene mutation<br>Test type: filtered out<br>Type of method: in Vitro<br>Test guideline: OECD Guideline 471 (Bacterial Reverse Mutation Assay)<br>Year: 1987<br>Title: Unnamed<br>Database: ECHA REACH	Test organisms (species): S. typhimurium TA 1538, TA 100, TA 98, TA 97, TA 1537, TA 1535,<br>Endpoint: Gene mutation<br>Test type: filtered out<br>Type of method: in Vitro<br>Year: 1982<br>Title: Unnamed<br>Database: ECHA REACH	Test organisms (species): Salmonella typhimurium / Escherichia coli<br>Endpoint: Gene mutation<br>Test type: Bacterial Reverse Mutation Assay (e.g. Ames Test)<br>Type of method: in Vitro<br>Strain: Salmonella typhimurium: TA 97, TA 1537, TA 1535, TA 100; Escherichia coli: WP2 UvrA<br>Test guideline: Guidelines For Screening Mutagenicity Testing of Chemicals

		Database: Genotoxicity OASIS	Database: Genotoxicity OASIS			(Chemical Substances Control Law of Japan)<br>Reference source: http://dra4.nihs.go.jp/mhlw_data/jsp/SearchPageENG.jsp<br>Author: Japan Existing Chemical Data Base<br>Title: Japan Ministry of Health Labour and Welfare<br>Database: Toxicity Japan MHLW
Value		Negative	Negative	Negative	Negative	Negative
Test description					Test organisms (species): S. typhimurium TA 100, TA 98, TA 1537, TA 1535<br>Endpoint: Gene mutation<br>Test type: filtered out<br>Type of method: in Vitro<br>Year: 1993<br>Title: Unnamed<br>Database: ECHA REACH	Test organisms (species): S. typhimurium TA 1535, TA 1537, TA 98, TA 100 and E. coli WP2<br>Endpoint: Gene mutation<br>Test type: filtered out<br>Type of method: in Vitro<br>Test guideline: OECD Guideline 471 (Bacterial Reverse Mutation Assay)<br>Year: 2006<br>Title: Unnamed<br>Database: ECHA REACH
Value					Negative	Negative
Test description					Test organisms (species): Chinese hamster Ovary (CHO)<br>Endpoint: in vitro gene mutation study in mammalian cells<br>Test type: filtered out<br>Type of method: in Vitro<br>Year: 2012<br>Title: Unnamed<br>Database: ECHA REACH	Test organisms (species): mouse lymphoma L5178Y cells<br>Endpoint: in vitro gene mutation study in mammalian cells<br>Test type: filtered out<br>Type of method: in Vitro<br>Year: 2013<br>Title: Unnamed<br>Database: ECHA REACH
Value					Negative	Negative
Endpoint		Chromosome aberration				

Test description				Test organisms (species): Chinese hamster Ovary (CHO)<br>Endpoint: in vitro cytogenicity / chromosome aberration study in mammalian cells<br>Test type: filtered out<br>Type of method: in Vitro<br>Test guideline: OECD Guideline 473 (In Vitro Mammalian Chromosome Aberration Test)<br>Year: 1987<br>Title: Unnamed<br>Database: ECHA REACH	Test organisms (species): Chinese hamster lung fibroblasts (V79)<br>Endpoint: in vitro cytogenicity / chromosome aberration study in mammalian cells<br>Test type: filtered out<br>Type of method: in Vitro<br>Year: 2012<br>Title: Unnamed<br>Database: ECHA REACH	Test organisms (species): Chinese hamster<br>Endpoint: Chromosome aberration<br>Test type: in Vitro Mammalian Chromosome Aberration Test<br>Type of method: in Vitro<br>Strain: Chinese Hamster Lung Cells<br>Test guideline: Guidelines For Screening Mutagenicity Testing of Chemicals (Chemical Substances Control Law of Japan)<br>Reference source: http://dra4.nihs.go.jp/mhlw_data/jsp/SearchPageENG.jsp<br>Title: Japan Ministry of Health Labour and Welfare<br>Database: Toxicity Japan MHLW
Value				Negative	Negative	negative
Test description				Test organisms (species): Other Test organisms (species)<br>Endpoint: in vitro DNA damage and/or repair study<br>Test type: filtered out<br>Type of method: in Vitro<br>Test guideline: OECD Guideline 482 (Genetic Toxicology: DNA Damage and Repair, Unscheduled DNA Synthesis in Mammalian Cells in Vitro)<br>Year: 1987<br>Title: Unnamed<br>Database: ECHA REACH		Test organisms (species): Chinese hamster lung fibroblasts (V79)<br>Endpoint: in vitro cytogenicity / chromosome aberration study in mammalian cells<br>Test type: filtered out<br>Type of method: in Vitro<br>Test guideline: OECD Guideline 473 (In Vitro Mammalian Chromosome Aberration Test)<br>Year: 2006<br>Title: Unnamed<br>Database: ECHA REACH

Value				Negative		Negative
Test description				Test organisms (species): mouse<br>Endpoint: in vivo mammalian somatic cell study: cytogenicity / erythrocyte micronucleus<br>Test type: filtered out<br>Type of method: in Vivo<br>Strain: CD-1<br>Year: 1993<br>Title: Unnamed<br>Database: ECHA REACH		
Value				Negative		

Table S 2, continued

**Substance**	**SD 3**	Analogue #1	Analogue #2	Analogue #3
**Substance identity**				
Structure	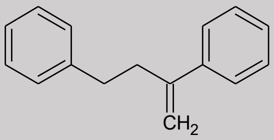	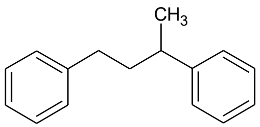	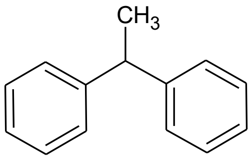	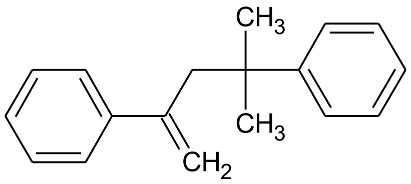
CAS number	16606-47-6	1520-44-1	38888-98-1	6362-80-7
Chemical name	2,4-Diphenyl-1-butene	1,3-diphenylbutane	Diphenylethane	2,4-Diphenyl-4-methylpentene-1
SMILES	C=C(CCc1ccccc1)c1ccccc1	CC(CCc1ccccc1)c1ccccc1	CC(c1ccccc1)c1ccccc1	CC(C)(CC(=C)c1ccccc1)c1ccccc1
**Profiles used for grouping/subcategorization**				
Structure similarity (primary grouping)	[90%,100%]	[60%,70%)	[70%,80%)	[70%,80%)
DNA binding by OECD (subcategorization)	Michael addition >> P450 Mediated Activation to Quinones and Quinone-type Chemicals >> Arenes			
Chemical elements (subcategorization)	Group 14 - Carbon C			
Structure similarity (subcategorization)	[90%,100%]	[60%,70%)	[70%,80%)	[70%,80%)
**Predictions**				
Sublevel	Genetic Toxicity			
Endpoint	Gene mutation <OR> in vitro cytogenicity / chromosome aberration study in mammalian cells <OR> in vitro DNA damage and/or repair study <OR>			

	in vitro gene mutation study in mammalian cells <OR> in vivo mammalian somatic cell study: cytogenicity / erythrocyte micronucleus			
Unit	Gene mutation I			
Reference	QSAR Toolbox Database: ECHA REACH <OR> Genotoxicity OASIS <OR> Toxicity Japan MHLW			
Prediction approach	Read-across			
Value	**negative**			
**Data used for prediction**				
Sublevel		Genetic Toxicity		
Endpoint		Gene Mutation		
Test description		Test organisms (species): Salmonella typhimurium<br>Endpoint: Gene mutation<br>Test type: Bacterial Reverse Mutation Assay (e.g. Ames Test)<br>Type of method: in Vitro<br>Strain: No Strain Info<br>Year: 1/1/2009 12:00:00 AM<br>Reference source: J. Chem. Inf. Model., 2009, 49 (9), pp 2077–2081<br>Author: Hansen K. et al.<br>Database: Genotoxicity OASIS	Test organisms (species): Other Test organisms (species)<br>Endpoint: Gene mutation<br>Test type: filtered out<br>Type of method: in Vitro<br>Test guideline: OECD Guideline 471 (Bacterial Reverse Mutation Assay)<br>Year: 1987<br>Title: Unnamed<br>Database: ECHA REACH	Test organisms (species): Salmonella typhimurium / Escherichia coli<br>Endpoint: Gene mutation<br>Test type: Bacterial Reverse Mutation Assay (e.g. Ames Test)<br>Type of method: in Vitro<br>Strain: Salmonella typhimurium: TA 97, TA 1537, TA 1535, TA 100; Escherichia coli: WP2 UvrA<br>Test guideline: Guidelines For Screening Mutagenicity Testing of Chemicals (Chemical Substances Control Law of Japan)<br>Reference source: http://dra4.nihs.go.jp/mhlw_data/jsp/SearchPageENG.jsp<br>Author: Japan Existing Chemical Data Base<br>Title: Japan Ministry of Health Labour and Welfare<br>Database: Toxicity Japan MHLW
Value		Negative	Negative	Negative

Test description				Test organisms (species): S. typhimurium TA 1535, TA 1537, TA 98, TA 100 and E. coli WP2<br>Endpoint: Gene mutation<br>Test type: filtered out<br>Type of method: in Vitro<br>Test guideline: OECD Guideline 471 (Bacterial Reverse Mutation Assay)<br>Year: 2006<br>Title: Unnamed<br>Database: ECHA REACH
Value				Negative
Test description				Test organisms (species): mouse lymphoma L5178Y cells<br>Endpoint: in vitro gene mutation study in mammalian cells<br>Test type: filtered out<br>Type of method: in Vitro<br>Year: 2013<br>Title: Unnamed<br>Database: ECHA REACH
Value				Negative
Endpoint		Chromosome aberration		
Test description			Test organisms (species): Chinese hamster Ovary (CHO)<br>Endpoint: in vitro cytogenicity / chromosome aberration study in mammalian cells<br>Test type: filtered out<br>Type of method: in Vitro<br>Test guideline: OECD Guideline 473 (In Vitro Mammalian Chromosome Aberration Test)<br>Year: 1987<br>Title: Unnamed<br>Database: ECHA REACH	Test organisms (species): Chinese hamster<br>Endpoint: Chromosome aberration<br>Test type: in Vitro Mammalian Chromosome Aberration Test<br>Type of method: in Vitro<br>Strain: Chinese Hamster Lung Cells<br>Test guideline: Guidelines For Screening Mutagenicity Testing of Chemicals (Chemical Substances Control Law of Japan)<br>Reference source: http://dra4.nihs.go.jp/mhlw_data/jsp/SearchPageENG.jsp<br>Title: Japan Ministry of Health Labour and Welfare<br>Database: Toxicity Japan MHLW
Value			Negative	negative

Test description			Test organisms (species): Other Test organisms (species)<br>Endpoint: in vitro DNA damage and/or repair study<br>Test type: filtered out<br>Type of method: in Vitro<br>Test guideline: OECD Guideline 482 (Genetic Toxicology: DNA Damage and Repair, Unscheduled DNA Synthesis in Mammalian Cells in Vitro)<br>Year: 1987<br>Title: Unnamed<br>Database: ECHA REACH	Test organisms (species): Chinese hamster lung fibroblasts (V79)<br>Endpoint: in vitro cytogenicity / chromosome aberration study in mammalian cells<br>Test type: filtered out<br>Type of method: in Vitro<br>Test guideline: OECD Guideline 473 (In Vitro Mammalian Chromosome Aberration Test)<br>Year: 2006<br>Title: Unnamed<br>Database: ECHA REACH
Value			Negative	Negative
Test description			Test organisms (species): mouse<br>Endpoint: in vivo mammalian somatic cell study: cytogenicity / erythrocyte micronucleus<br>Test type: filtered out<br>Type of method: in Vivo<br>Strain: CD-1<br>Year: 1993<br>Title: Unnamed<br>Database: ECHA REACH	
Value			Negative	

Table S 2, continued

**Substance**	**SD 4**	Analogue #1	Analogue #2	Analogue #3
**Substance identity**				
Structure	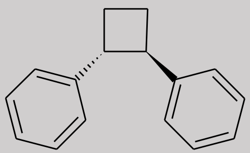	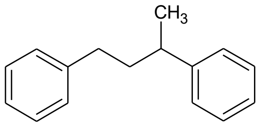	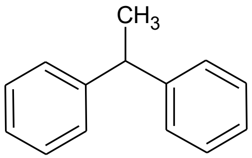	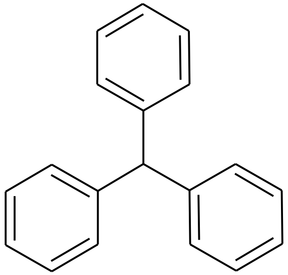
CAS number	20071-09-4	1520-44-1	38888-98-1	519-73-3
Chemical name	trans -1,2-diphenylcyclobutan	1,3-diphenylbutane	Diphenylethane	triphenylmethane
SMILES	C1C[C@H](C1c1ccccc1)c1ccccc1	CC(CCc1ccccc1)c1ccccc1	CC(c1ccccc1)c1ccccc1	c1ccc(cc1)C(c1ccccc1)c1ccccc1
**Profiles used for grouping/subcategorization**				
Structure similarity (primary grouping)	[90%,100%]	[80%,90%)	[70%,80%)	[60%,70%)
DNA binding by OECD (subcategorization)	Michael addition >> P450 Mediated Activation to Quinones and Quinone-type Chemicals >> Arenes			
Chemical elements (subcategorization)	Group 14 - Carbon C			
Substance type (subcategorization)	Discrete chemical; Mono constituent (predefined); Organic	Discrete chemical; Mono constituent (predefined); Organic	Discrete chemical; Mono constituent (predefined); Organic	Discrete chemical; Mono constituent (predefined); Organic
Structure similarity (subcategorization)	[90%,100%]	[80%,90%)	[70%,80%)	[60%,70%)

**Predictions**				
Sublevel	Genetic Toxicity			
Endpoint	Gene mutation <OR> in vitro cytogenicity / chromosome aberration study in mammalian cells <OR> in vitro DNA damage and/or repair study <OR> in vitro gene mutation study in mammalian cells <OR> in vivo mammalian somatic cell study: cytogenicity / erythrocyte micronucleus			
Unit	Gene mutation I			
Reference	QSAR Toolbox Database: ECHA REACH <OR> Genotoxicity OASIS <OR> Toxicity Japan MHLW			
Prediction approach	Read-across			
Value	**negative**			
**Data used for prediction**				
Sublevel		Genetic Toxicity		
Endpoint		Gene Mutation		
Test description		Test organisms (species): Salmonella typhimurium<br>Endpoint: Gene mutation<br>Test type: Bacterial Reverse Mutation Assay (e.g. Ames Test)<br>Type of method: in Vitro<br>Strain: No Strain Info<br>Year: 1/1/2009 12:00:00 AM<br>Reference source: J. Chem. Inf. Model., 2009, 49 (9), pp 2077–2081<br>Author: Hansen K. et al.<br>Database: Genotoxicity OASIS	Test organisms (species): Other Test organisms (species)<br>Endpoint: Gene mutation<br>Test type: filtered out<br>Type of method: in Vitro<br>Test guideline: OECD Guideline 471 (Bacterial Reverse Mutation Assay)<br>Year: 1987<br>Title: Unnamed<br>Database: ECHA REACH	Test organisms (species): Salmonella typhimurium<br>Endpoint: Gene mutation<br>Test type: Bacterial Reverse Mutation Assay (e.g. Ames Test)<br>Type of method: in Vitro<br>Strain: No Strain Info<br>Reference source: Journal of Medicinal Chemistry 48 (1), pp. 312-32, 25<br>Author: Kazius J, McGuire R., Bursi R<br>Database: Genotoxicity OASIS

Value		Negative	Negative	negative
Test description				Test organisms (species): Salmonella typhimurium<br>Endpoint: Gene mutation<br>Test type: Bacterial Reverse Mutation Assay (e.g. Ames Test)<br>Type of method: in Vitro<br>Strain: TA 98, TA 97, TA 104, TA 100,<br>Year: 02.5.2011<br>Reference source: CCRIS database, Toxnet databases<br>Author: Romualdo Benigni<br>Database: Bacterial mutagenicity ISSSTY
Value				negative
Endpoint		Chromosome aberration		
Test description			Test organisms (species): Chinese hamster Ovary (CHO)<br>Endpoint: in vitro cytogenicity / chromosome aberration study in mammalian cells<br>Test type: filtered out<br>Type of method: in Vitro<br>Test guideline: OECD Guideline 473 (In Vitro Mammalian Chromosome Aberration Test)<br>Year: 1987<br>Title: Unnamed<br>Database: ECHA REACH	
Value			Negative	
Test description			Test organisms (species): Other Test organisms (species)<br>Endpoint: in vitro DNA damage and/or repair study<br>Test type: filtered out<br>Type of method: in Vitro<br>Test guideline: OECD Guideline 482 (Genetic Toxicology: DNA Damage and Repair, Unscheduled DNA Synthesis in Mammalian Cells in Vitro)<br>Year: 1987<br>Title: Unnamed<br>Database: ECHA REACH	

Value			Negative	
Test description			Test organisms (species): mouse<br>Endpoint: in vivo mammalian somatic cell study: cytogenicity / erythrocyte micronucleus<br>Test type: filtered out<br>Type of method: in Vivo<br>Strain: CD-1<br>Year: 1993<br>Title: Unnamed<br>Database: ECHA REACH	
Value			Negative	

Table S 2, continued

Substance	ST 1	Analogue #1	Analogue #2	Analogue #3	Analogue #4	Analogue #5
**Substance identity**						
Structure	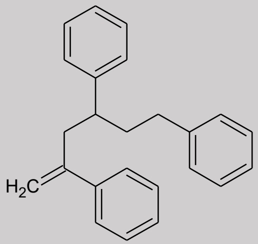	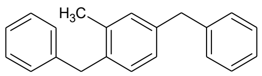	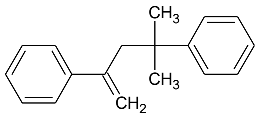	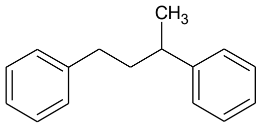	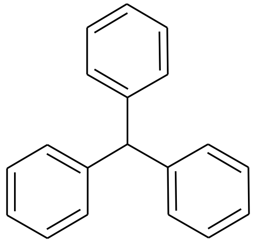	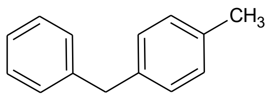
CAS number	18964-53-9	26898-17-9	6362-80-7	1520-44-1	519-73-3	27776-01-8
Chemical name	2,4,6- triphenyl-1-hexen	dibenzyltoluene	2,4-Diphenyl-4-methylpentene-1	1,3-diphenylbutane	triphenylmethane	Benzene, methyl(phenylmethyl)-
SMILES	C=C(CC(CCc1ccccc1)c1ccccc1)c1ccccc1	Cc1cc(Cc2ccccc2)ccc1Cc1ccccc1	CC(C)(CC(=C)c1ccccc1)c1ccccc1	Cc1cc(Cc2ccccc2)ccc1Cc1ccccc1	c1ccc(cc1)C(c1ccccc1)c1ccccc1	Cc1ccc(Cc2ccccc2)cc1
**Profiles used for grouping/subcategorization**						
Structure similarity (primary grouping)	[90%,100%]	[50%,60%)	[60%,70%)	[70%,80%)	[70%,80%)	[50%,60%)
DNA binding by OECD (subcategorization)	Michael addition >> P450 Mediated Activation to Quinones and Quinone-type Chemicals >> Arenes					
Chemical elements (subcategorization)	Group 14 - Carbon C					
Structure similarity (subcategorization)	[90%,100%]	[50%,60%)	[60%,70%)	[70%,80%)	[70%,80%)	[50%,60%)
**Predictions**						
Sublevel	Genetic Toxicity					

Endpoint	Gene mutation <OR> in vitro cytogenicity / chromosome aberration study in mammalian cells <OR> in vitro DNA damage and/or repair study <OR> in vitro gene mutation study in mammalian cells <OR> in vivo mammalian somatic cell study: cytogenicity / erythrocyte micronucleus					
Unit	Gene mutation I					
Reference	QSAR Toolbox Database: ECHA REACH <OR> Genotoxicity OASIS <OR> Toxicity Japan MHLW					
Prediction approach	Read-across					
Value	**negative**					
**Data used for prediction**						
Sublevel		Genetic Toxicity				
Endpoint		Gene Mutation				
Test description		Test organisms (species): Salmonella typhimurium / Escherichia coli<br>Endpoint: Gene mutation<br>Test type: Bacterial Reverse Mutation Assay (e.g. Ames Test)<br>Type of method: in Vitro<br>Strain: Salmonella typhimurium: TA 97, TA 1537, TA 1535, TA 100; Escherichia coli: WP2 UvrA<br>Test guideline: Guidelines For Screening	Test organisms (species): Salmonella typhimurium / Escherichia coli<br>Endpoint: Gene mutation<br>Test type: Bacterial Reverse Mutation Assay (e.g. Ames Test)<br>Type of method: in Vitro<br>Strain: Salmonella typhimurium: TA 97, TA 1537, TA 1535, TA 100; Escherichia coli: WP2 UvrA<br>Test guideline: Guidelines For Screening	Test organisms (species): Salmonella typhimurium<br>Endpoint: Gene mutation<br>Test type: Bacterial Reverse Mutation Assay (e.g. Ames Test)<br>Type of method: in Vitro<br>Strain: No Strain Info<br>Year: 1/1/2009 12:00:00 AM<br>Reference source: J. Chem. Inf. Model., 2009, 49 (9), pp 2077–2081<br>Author: Hansen K. et al.	Test organisms (species): Salmonella typhimurium<br>Endpoint: Gene mutation<br>Test type: Bacterial Reverse Mutation Assay (e.g. Ames Test)<br>Type of method: in Vitro<br>Strain: No Strain Info<br>Reference source: Journal of Medicinal Chemistry 48 (1), pp. 312-32, 25<br>Author: Kazius J, McGuire R., Bursi R<br>Database: Genotoxicity OASIS	Test organisms (species): Salmonella typhimurium<br>Endpoint: Gene mutation<br>Test type: Bacterial Reverse Mutation Assay (e.g. Ames Test)<br>Type of method: in Vitro<br>Strain: No Strain Info<br>Year: 1/1/2009 12:00:00 AM<br>Reference source: J. Chem. Inf. Model., 2009, 49 (9), pp 2077–2081<br>Author: Hansen K. et al.

		Mutagenicity Testing of Chemicals (Chemical Substances Control Law of Japan)<br>Reference source: http://dra4.nihs.go.jp/mhlw_data/jsp/SearchPageENG.jsp<br>Author: Japan Existing Chemical Data Base<br>Title: Japan Ministry of Health Labour and Welfare<br>Database: Toxicity Japan MHLW	Mutagenicity Testing of Chemicals (Chemical Substances Control Law of Japan)<br>Reference source: http://dra4.nihs.go.jp/mhlw_data/jsp/SearchPageENG.jsp<br>Author: Japan Existing Chemical Data Base<br>Title: Japan Ministry of Health Labour and Welfare<br>Database: Toxicity Japan MHLW	Database: Genotoxicity OASIS		Database: Genotoxicity OASIS
Value		Negative	Negative	negative	negative	negative
Test description			Test organisms (species): S. typhimurium TA 1535, TA 1537, TA 98, TA 100 and E. coli WP2<br>Endpoint: Gene mutation<br>Test type: filtered out<br>Type of method: in Vitro<br>Test guideline: OECD Guideline 471 (Bacterial Reverse Mutation Assay)<br>Year: 2006<br>Title: Unnamed<br>Database: ECHA REACH		Test organisms (species): Salmonella typhimurium<br>Endpoint: Gene mutation<br>Test type: Bacterial Reverse Mutation Assay (e.g. Ames Test)<br>Type of method: in Vitro<br>Strain: TA 98, TA 97, TA 104, TA 100,<br>Year: 02.5.2011<br>Reference source: CCRIS database, Toxnet databases<br>Author: Romualdo Benigni<br>Database: Bacterial mutagenicity ISSSTY	
Value			Negative		negative	
Test description			Test organisms (species): mouse lymphoma L5178Y cells<br>Endpoint: in vitro gene mutation study in mammalian cells<br>Test type: filtered out<br>Type of method: in Vitro			

			Year: 2013<br>Title: Unnamed<br>Database: ECHA REACH			
Value			Negative			
Endpoint		Chromosome aberration				
Test description		Test organisms (species): Chinese hamster<br>Endpoint: Chromosome aberration<br>Test type: in Vitro Mammalian Chromosome Aberration Test<br>Type of method: in Vitro<br>Strain: Chinese Hamster Lung Cells<br>Test guideline: Guidelines For Screening Mutagenicity Testing of Chemicals (Chemical Substances Control Law of Japan)<br>Reference source: http://dra4.nihs.go.jp/mhlw_data/jsp/SearchPageENG.jsp<br>Title: Japan Ministry of Health Labour and Welfare<br>Database: Toxicity Japan MHLW	Test organisms (species): Chinese hamster<br>Endpoint: Chromosome aberration<br>Test type: in Vitro Mammalian Chromosome Aberration Test<br>Type of method: in Vitro<br>Strain: Chinese Hamster Lung Cells<br>Test guideline: Guidelines For Screening Mutagenicity Testing of Chemicals (Chemical Substances Control Law of Japan)<br>Reference source: http://dra4.nihs.go.jp/mhlw_data/jsp/SearchPageENG.jsp<br>Title: Japan Ministry of Health Labour and Welfare<br>Database: Toxicity Japan MHLW			
Value		Negative	negative			
Test description			Test organisms (species): Chinese hamster lung fibroblasts (V79)<br>Endpoint: in vitro cytogenicity / chromosome aberration study in mammalian cells			

			Test type: filtered out<br>Type of method: in Vitro<br>Test guideline: OECD Guideline 473 (In Vitro Mammalian Chromosome Aberration Test)<br>Year: 2006<br>Title: Unnamed<br>Database: ECHA REACH			
Value			Negative			

Table S 2, continued

Substance	ST 2 – ST 5	Analogue #1	Analogue #2	Analogue #3	Analogue #4	Analogue #5
**Substance identity**						
Structure	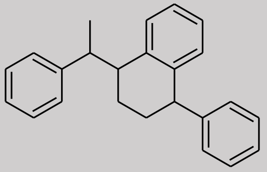	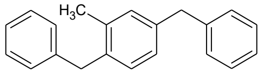	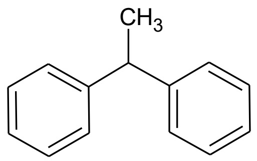	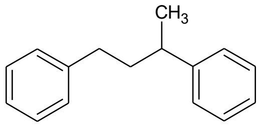	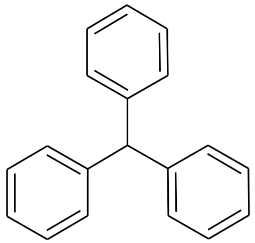	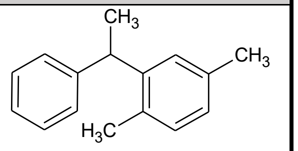
CAS number	26681-79-8	26898-17-9	38888-98-1	1520-44-1	519-73-3	6165-51-1
Chemical name	1a-phenyl-4a-(1-phenylethyl)-1,2,3,4 tetrahydronaphtale	dibenzyltoluene	Diphenylethane	1,3-diphenylbutane	triphenylmethane	2-(1-phenylethyl)-p-xylene
SMILES	CC(C1CCC(c2ccccc2)c2cccc c12)c1ccccc1	Cc1cc(Cc2ccccc2)ccc1Cc1c cccc1	CC(c1ccccc1)c1ccccc1	Cc1cc(Cc2ccccc2)ccc1Cc1c cccc1	c1ccc(cc1)C(c1ccccc1)c1cc ccc1	CC(c1ccccc1)c1cc(C)ccc1C
**Profiles used for grouping/subcategorization**						
Structure similarity (primary grouping)	[90%,100%]	[50%,60%)	[60%,70%)	[60%,70%)	[70%,80%)	[50%,60%)
DNA binding by OECD (subcategorization)	Michael addition >> P450 Mediated Activation to Quinones and Quinone-type Chemicals >> Arenes					
Chemical elements (subcategorization)	Group 14 - Carbon C					
Structure similarity (subcategorization)	[90%,100%]	[50%,60%)	[60%,70%)	[60%,70%)	[70%,80%)	[50%,60%)
**Predictions**						
Sublevel	Genetic Toxicity					

Endpoint	Gene mutation <OR> in vitro cytogenicity / chromosome aberration study in mammalian cells <OR> in vitro DNA damage and/or repair study <OR> in vitro gene mutation study in mammalian cells <OR> in vivo mammalian somatic cell study: cytogenicity / erythrocyte micronucleus					
Unit	Gene mutation I					
Reference	QSAR Toolbox Database: ECHA REACH <OR> Genotoxicity OASIS <OR> Toxicity Japan MHLW					
Prediction approach	Read-across					
Value	**negative**					
**Data used for prediction**						
Sublevel		Genetic Toxicity				
Endpoint		Gene Mutation				
Test description		Test organisms (species): Salmonella typhimurium / Escherichia coli<br>Endpoint: Gene mutation<br>Test type: Bacterial Reverse Mutation Assay (e.g. Ames Test)<br>Type of method: in Vitro<br>Strain: Salmonella typhimurium: TA 97, TA 1537, TA 1535, TA 100; Escherichia coli: WP2 UvrA<br>Test guideline: Guidelines For Screening	Test organisms (species): Other Test organisms (species)<br>Endpoint: Gene mutation<br>Test type: filtered out<br>Type of method: in Vitro<br>Test guideline: OECD Guideline 471 (Bacterial Reverse Mutation Assay)<br>Year: 1987<br>Title: Unnamed<br>Database: ECHA REACH	Test organisms (species): Salmonella typhimurium<br>Endpoint: Gene mutation<br>Test type: Bacterial Reverse Mutation Assay (e.g. Ames Test)<br>Type of method: in Vitro<br>Strain: No Strain Info<br>Year: 1/1/2009 12:00:00 AM<br>Reference source: J. Chem. Inf. Model., 2009, 49 (9), pp 2077–2081<br>Author: Hansen K. et al.	Test organisms (species): Salmonella typhimurium<br>Endpoint: Gene mutation<br>Test type: Bacterial Reverse Mutation Assay (e.g. Ames Test)<br>Type of method: in Vitro<br>Strain: No Strain Info<br>Reference source: Journal of Medicinal Chemistry 48 (1), pp. 312-32, 25<br>Author: Kazius J, McGuire R., Bursi R<br>Database: Genotoxicity OASIS	Test organisms (species): Salmonella typhimurium / Escherichia coli<br>Endpoint: Gene mutation<br>Test type: Bacterial Reverse Mutation Assay (e.g. Ames Test)<br>Type of method: in Vitro<br>Strain: Salmonella typhimurium: TA 97, TA 1537, TA 1535, TA 100; Escherichia coli: WP2 UvrA<br>Test guideline: Guidelines For Screening Mutagenicity Testing of Chemicals

		Mutagenicity Testing of Chemicals (Chemical Substances Control Law of Japan)<br>Reference source: http://dra4.nihs.go.jp/mhlw_data/jsp/SearchPageENG.jsp<br>Author: Japan Existing Chemical Data Base<br>Title: Japan Ministry of Health Labour and Welfare<br>Database: Toxicity Japan MHLW		Database: Genotoxicity OASIS		(Chemical Substances Control Law of Japan)<br>Reference source: http://dra4.nihs.go.jp/mhlw_data/jsp/SearchPageENG.jsp<br>Author: Japan Existing Chemical Data Base<br>Title: Japan Ministry of Health Labour and Welfare<br>Database: Toxicity Japan MHLW
Value		Negative	Negative	Negative	negative	negative
Test description					Test organisms (species): Salmonella typhimurium<br>Endpoint: Gene mutation<br>Test type: Bacterial Reverse Mutation Assay (e.g. Ames Test)<br>Type of method: in Vitro<br>Strain: TA 98, TA 97, TA 104, TA 100,<br>Year: 02.5.2011<br>Reference source: CCRIS database, Toxnet databases<br>Author: Romualdo Benigni<br>Database: Bacterial mutagenicity ISSSTY	Test organisms (species): Salmonella typhimurium<br>Endpoint: Gene mutation<br>Test type: Bacterial Reverse Mutation Assay (e.g. Ames Test)<br>Type of method: in Vitro<br>Strain: No Information For Experimental Cells<br>Reference source: NIHS Japan<br>Author: NIHS Japan<br>Database: Genotoxicity OASIS
Value					negative	negative
Test description						Test organisms (species): Salmonella typhimurium / Escherichia coli<br>Endpoint: Gene mutation<br>Test type: Bacterial Reverse Mutation Assay (e.g. Ames Test)<br>Type of method: in Vitro

						Strain: S. Typhimurium: TA 100, TA 98, TA 1537, TA 1535; Escherichia coli: WP2 UvrA<br>Test guideline: OECD Guideline 471 (Bacterial Reverse Mutation Assay)<br>Reference source: Japan Existing Chemical Data Base; http://dra4.nihs.go.jp/mhlw_data/jsp/SearchPageENG.jsp<br>Author: Japan Existing Chemical Data Base; http://dra4.nihs.go.jp/mhlw_data/jsp/SearchPageENG.jsp<br>Database: Genotoxicity OASIS
Value						negative
Endpoint		Chromosome aberration				
Test description			Test organisms (species): Chinese hamster Ovary (CHO)<br>Endpoint: in vitro cytogenicity / chromosome aberration study in mammalian cells<br>Test type: filtered out<br>Type of method: in Vitro<br>Test guideline: OECD Guideline 473 (In Vitro Mammalian Chromosome Aberration Test)<br>Year: 1987<br>Title: Unnamed<br>Database: ECHA REACH			Test organisms (species): Chinese hamster<br>Endpoint: Chromosome aberration<br>Test type: in Vitro Mammalian Chromosome Aberration Test<br>Type of method: in Vitro<br>Strain: Chinese Hamster Lung Cells<br>Test guideline: Guidelines For Screening Mutagenicity Testing of Chemicals (Chemical Substances Control Law of Japan)<br>Reference source: http://dra4.nihs.go.jp/mhlw_data/jsp/SearchPageENG.jsp

						Title: Japan Ministry of Health Labour and Welfare<br>Database: Toxicity Japan MHLW
Value			Negative			negative
Test description			Test organisms (species): Other Test organisms (species)<br>Endpoint: in vitro DNA damage and/or repair study<br>Test type: filtered out<br>Type of method: in Vitro<br>Test guideline: OECD Guideline 482 (Genetic Toxicology: DNA Damage and Repair, Unscheduled DNA Synthesis in Mammalian Cells in Vitro)<br>Year: 1987<br>Title: Unnamed<br>Database: ECHA REACH			Endpoint: Chromosome aberration<br>Test type: in Vitro Mammalian Chromosome Aberration Test<br>Type of method: in Vitro<br>Strain: No Strain Information<br>Reference source: NIHS Japan<br>Author: NIHS Japan<br>Database: Genotoxicity OASIS
Value			Negative			Negative
Test description			Test organisms (species): mouse<br>Endpoint: in vivo mammalian somatic cell study: cytogenicity / erythrocyte micronucleus<br>Test type: filtered out<br>Type of method: in Vivo<br>Strain: CD-1<br>Year: 1993<br>Title: Unnamed<br>Database: ECHA REACH			
Value			Negative			

Table S 3 Results of the genotoxicity prediction for styrene oligomers and SD 4-analogues using DEREK Nexus and SARAH Nexus

Compound	Result Mutagenicity in vitro in bacterium DEREK Nexus	Misclassified or unclassified features?	Explanation of the result	Mutagenicity prediction SARAH Nexus	Confidence
**SD 1**	inactive	no	The query structure does not match any structural alerts or examples in DEREK which show activity in a bacterial reverse mutation assay (Ames test). Additionally, the query structure does not contain any unclassified or misclassified features.	negative	56%
**SD 3**	inactive	no		negative	40%
**SD 4**	inactive	no		equivocal	-
**ST 1**	inactive	no		negative	37%
**ST 2 - ST 5**	inactive	no		negative	16%

SD 4 analogues:

No	SMILES	Structure	Result Mutagenicity in vitro in bacterium DEREK Nexus	Misclassified or unclassified features?	Explanation of the result	Mutagenicity prediction SARAH Nexus	Confidence
1	C1CCC1	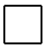	inactive	no	The query structure does not match any structural alerts or examples in DEREK which show activity in a bacterial reverse mutation assay (Ames test). Additionally, the query structure does not contain any unclassified or misclassified features.	equivocal	-
2	c1cccc(c1) C1CCC1	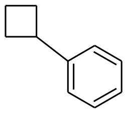	inactive	no		equivocal	-
3	CC1CCC1c 1ccccc1	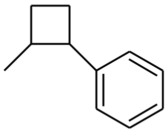	inactive	no		negative	26%
4	CC1(CCC1) c1ccccc1	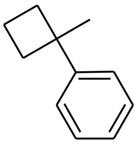	inactive	no		negative	26%
5	CC1CC(C1) c1ccccc1	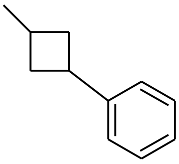	inactive	no		negative	26%
6	CC1(CC(C)(C1)c1ccccc 1)c1ccccc1	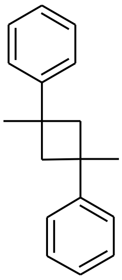	inactive	no		negative	23%

Table S 4 Results of the genotoxicity prediction for styrene oligomers and SD 4-analogues using the Danish (DTU) QSAR database

Compound	Result Bacterial reverse mutation test (Ames) in S. typhimurium (in vitro)	Probability	Inside applicability domain?	Result Chromosome Aberrations in Chinese Hamster Lung Cells	Probability	Inside applicability domain?
**SD 1**	negative	0.141	yes	negative	0.111	yes
**SD 3**	negative	0.061	yes	negative	0.111	yes
**SD 4**	negative	0.359	no	inconclusive	-	no
**ST 1**	negative	0.082	yes	negative	0.089	yes
**ST 2 - ST 5**	negative	0.29	yes	negative	0.004	yes

SD 4 analogues:

No	SMILES	Structure	Result Bacterial reverse mutation test (Ames) in S. typhimurium (in vitro)	Probability	Inside applicability domain?	Result Chromosome Aberrations in Chinese Hamster Lung Cells	Probability	Inside applicability domain?
1	C1CCC1	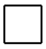	inconclusive	-	no	inconclusive	-	no
2	c1cccc(c1)C1CCC1	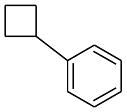	negative	0.687	no	inconclusive	-	no
3	CC1CCC1c1ccccc1	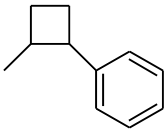	negative	0.678	no	inconclusive	-	no
4	CC1(CCC1)c1ccccc1	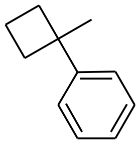	inconclusive	-	no	inconclusive	-	no
5	CC1CC(C1)c1ccccc1	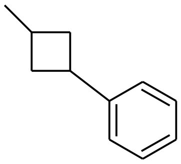	negative	0.678	no	inconclusive	-	no
6	CC1(CC(C)(C1)c1ccccc1)c1ccccc1	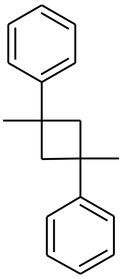	negative	0.718	yes	inconclusive	-	no
